# Multi-Objective Community Detection Based on Memetic Algorithm

**DOI:** 10.1371/journal.pone.0126845

**Published:** 2015-05-01

**Authors:** Peng Wu, Li Pan

**Affiliations:** 1 School of Electronic Information and Electrical Engineering, Shanghai Jiao Tong University, Shanghai, China; 2 National Engineering Laboratory for Information Content Analysis Technology, Shanghai Jiao Tong University, Shanghai, China; University Toulouse 1 Capitole, FRANCE

## Abstract

Community detection has drawn a lot of attention as it can provide invaluable help in understanding the function and visualizing the structure of networks. Since single objective optimization methods have intrinsic drawbacks to identifying multiple significant community structures, some methods formulate the community detection as multi-objective problems and adopt population-based evolutionary algorithms to obtain multiple community structures. Evolutionary algorithms have strong global search ability, but have difficulty in locating local optima efficiently. In this study, in order to identify multiple significant community structures more effectively, a multi-objective memetic algorithm for community detection is proposed by combining multi-objective evolutionary algorithm with a local search procedure. The local search procedure is designed by addressing three issues. Firstly, nondominated solutions generated by evolutionary operations and solutions in dominant population are set as initial individuals for local search procedure. Then, a new direction vector named as pseudonormal vector is proposed to integrate two objective functions together to form a fitness function. Finally, a network specific local search strategy based on label propagation rule is expanded to search the local optimal solutions efficiently. The extensive experiments on both artificial and real-world networks evaluate the proposed method from three aspects. Firstly, experiments on influence of local search procedure demonstrate that the local search procedure can speed up the convergence to better partitions and make the algorithm more stable. Secondly, comparisons with a set of classic community detection methods illustrate the proposed method can find single partitions effectively. Finally, the method is applied to identify hierarchical structures of networks which are beneficial for analyzing networks in multi-resolution levels.

## Introduction

Many real-world systems which consist of objects with relationships among them can be efficaciously represented as complex networks [1]. Community structure is one of the most important properties of diverse networks. Generally speaking, a community can be described as a group of nodes that are densely intra-connected, while only sparsely linked with the rest of the network [2]. Community structures in complex networks play important roles in the structure-function relationship, as they can provide invaluable help in understanding the functions and visualizing the structures of networks [3, 4].

Most community detection methods can be roughly classified into heuristic based and optimization based methods. Heuristic based methods derive network partitions by executing some heuristic rules which are usually based on intuitive observations rather than explicitly optimizing global objective functions [5]. Such kind of methods usually lacks of accurate description of the properties of global community structures. Optimization based methods formulate community detection as a combinatorial optimization problem and detect the community structure by optimizing a predefined evaluation criterion which describes a certain property of community, such as modularity [6], normalized cut [7] and the map equation [8], etc. However, single objective optimization methods have two main intrinsic disadvantages, i.e., they may lead to bias on the obtained community partition, and they may not be able to detect multiple potential structures [9]. To overcome above drawbacks, community detection problems have also been formulated as Multi-objective Optimization Problems (MOPs). Multi-objective community detection methods describe multiple structure properties of networks by optimizing multiple conflicting criteria and obtain multiple network partitions which correspond to different tradeoffs among these criteria [10, 11].

Traditional community detection methods dealing with single objective function and single community partition can be hardly adapted to multi-objective community detection problems, so Evolutionary Algorithms (EAs) which can handle a population of partitions in a single run have been adopted [9, 12, 13]. EAs have excellent global search abilities of exploring the entire network partition space and identifying areas with potential high quality partitions. However, they have difficulty in locating the local best partitions around the potential high quality space in a short time. To address such drawbacks, Memetic Algorithms (MAs) which combine EAs with local search procedure have been proposed to deal with single objective community detection problems so far [14–17]. The integrated local search procedure can search the promising partition space carefully and accelerate the method to find the local optimal partitions. The hybrid search property of MAs should have advantages to deal with multi-objective community detection problems too. Since conventional local search methods only optimize single fitness function and deal with one partition at a time, three problems need to be addressed to adapt them to multi-objective situations, i.e., determining initial partitions, defining appropriate fitness function and designing effective local search strategy.

In this paper, a multi-objective memetic community detection algorithm to identify multiple community structures is presented. The proposed algorithm is termed as MMCD for short. The MMCD adopts a multi-objective immune algorithm as global search mechanism. We mainly focus our effort on developing an effective local search procedure by addressing three problems. Firstly, nondominated solutions generated by evolutionary operations and solutions in dominant population are selected as initial partitions for local search procedure. Then, a new direction vector named as pseudonormal vector is proposed to integrate two objective functions together to form a fitness function. Finally, a network specific local search strategy is expanded to search the local optimal solutions efficiently. To evaluate the effect of local search procedure on MMCD and illustrate the important applications of MMCD, experiments on artificial datasets and real-world networks are carried out from three perspectives, i.e. parameter settings and effects of local search procedure, comparison with a variety of community detection methods, and ability to find multi-resolution structures of networks.

The remainder of this paper is organized as follows. Section 2 presents works related to our work. Section 3 describes the formulation of multi-objective optimization community detection problem. The method is described in section 4. Section 5 presents the experimental results. Finally, Section 6 gives the conclusions.

## Related Works

Some multi-objective optimization community detection methods have been proposed based on EAs which are inspired by biological evolution. The Multi-objective Genetic Algorithm for Networks (MOGA-Net) [12] optimizes community score and community fitness simultaneously. It adopts the Nondominated Sorting Genetic Algorithm-II (NSGA-II) [18] as optimization mechanism. The Multi-Objective Community Detection algorithm (MOCD) [13] selects two terms of modularity as objective functions. Since two terms of modularity describe two opposite properties of a community structure and are appropriate for detecting multiple structures, we also adopt them as conflicting objective functions in this paper. However, the optimization algorithm proposed in this paper is rather different from MOCD. MOCD adopts the Pareto Envelope-based Selection Algorithm version 2 (PESA-II) [19] as optimization mechanism and does not integrate a local search procedure, while our method integrates a local search procedure into another competitive multi-objective EA, i.e. Nondominated Neighbor Immune Algorithm (NNIA) [20]. The NNIA is also adopted by Multi-objective Immune algorithm for multi-resolution Community Detection (MICD) [9]. However, MICD adopts two terms of modularity density as objective functions and also does not integrate a local search procedure. What’s more, the individual representation scheme and the evolution operators used by MMCD are different from those used by MOCD and MICD.

One of the biggest differences between MMCD and other multi-objective community detection algorithms is that it integrates a local search procedure into the multi-objective EA. Conventional EAs which search the community partition space based on random evolution without much restriction have strong global search ability [9, 12, 21, 22]. However, they have difficulty in locating the local optimal solutions around the promising search space in a short time. On the other hand, local search community detection methods are very good at obtaining the local best partitions [23–25], while the salient drawback is that escaping from the local optima to achieve a better solution is not easy for them. Based on advantages and drawbacks mentioned above, it is profitable to combine EAs and local search methods together to formulate the Memetic Algorithms for community detection problems. For example, the Meme-Net community detection algorithm combines GAs with a hill-climbing local search strategy to optimize the modularity density [14]. The community detection method based on Modularity and an Improved Genetic Algorithm (MIGA) takes modularity as objective function and adopts simulated annealing method as local search method [15]. The Memetic algorithm with multi-level Learning strategies (MLCD) also optimizes modularity and proposes multi-level learning methods to accelerate the convergence of genetic algorithm [16]. Above MAs for community detection mainly optimize single objective functions. It is valuable to extend MAs to handle multi-objective community detection problems due to the advantages of multi-objective formulations.

## Problem Formulations

In this paper, a network *N* is modeled as a graph *G* = (*V*,*E*) with adjacency matrix *A*, where *V* and *E* are the sets of nodes and edges respectively. A community partition is encoded as *X* = (*x*_1_, *x*_2_, …, *x*_*n*_) where *x*_*i*_ is the code value of node *i*. A multi-objective maximization community detection problem [9, 12] (Ω, *f*_1_, *f*_2_,…, *f*_*t*_) is formulated as

$$\begin{array}{c} \max F\left(X\right)={\left(f_{1}\left(X\right),f_{2}\left(X\right),\ldots ,f_{t}\left(X\right)\right)}^{T},\;\text{subject}\;\text{to}\;\;X=\left(x_{1},x_{2},\ldots ,x_{n}\right)\in \Omega , \end{array}$$

where Ω = (*X*_1_, *X*_2_,…, *X*_*N*_) is the set of all feasible partitions of a network, and *f*_1_, *f*_2_,…, *f*_*t*_ denote different objective functions which describe the properties of the partition obtained. To define the set of solutions which are found by the use of Pareto optimality theory [26], the definition of dominance relation is given below. Given any two feasible solutions *X*_1_ and *X*_2_, solution *X*_1_ is said to dominate solution *X*_2_, denoted as *X*_1_ ≻ *X*_2_, iff

$$\begin{array}{c} \forall \;i=1,2,\ldots ,t:f_{i}\left(X_{1}\right)\geq f_{i}\left(X_{2}\right)\;\wedge \;\exists \;i=1,2,\ldots ,t:f_{i}\left(X_{1}\right)>f_{i}\left(X_{2}\right). \end{array}$$

*X*_2_ is called dominated solution. If there does not exist any other solution *X* that dominates X*, X* is called nondominated (i.e. dominant) or Pareto-optimal solution. Pareto-optimal solutions do not dominate each other, as an improvement in one objective function will result in a degradation of another. The set of Pareto-optimal solutions is called the Pareto-optimal set which is formulated as

$$\begin{array}{c} \mathrm{PS}≜\{X^{*}\in \Omega |∄X\in \Omega ,X≻X^{*}\}. \end{array}$$

The functions vector *F* maps the solution space into the objective function space, and the corresponding image of the Pareto-optimal set in the objective function space is called the Pareto-optimal front which is formulated as

$$\begin{array}{c} \mathrm{PF}\;≜\;\{F\left(X^{*}\right)\;=\;{\left(f_{1}\left(X^{*}\right),f_{2}\left(X^{*}\right),\ldots ,f_{t}\left(X^{*}\right)\right)}^{T}|X^{*}\;\in \;\mathrm{PS}\}. \end{array}$$

The goal of the multi-objective community detection methods is to find a Pareto-optimal set or an approximated one.

Since communities are usually described from two aspects that they are usually densely intra-connected and sparsely inter-connected, choosing two objective functions to reflect such two properties is reasonable. In this paper, two parts of modularity are selected as two objective functions because they can reflect such two properties to some extent.

Modularity (denoted as *Q*) proposed by Girvan and Newman [6] can be written as

$$\begin{array}{c} Q=\sum_{C\in X}[\frac{l_{C}}{m}-{\left(\frac{k_{C}}{2m}\right)}^{2}], \end{array}$$ (1)

where *X* is one possible partition of the network, *C* is a community in partition *X*, *l*_*C*_ is the number of edges in the community *C* which is defined as *l*_*C*_ = (∑_*i*, *j* ∈ *C*_ *A*_*ij*_)/2, *k*_*C*_ is the total degree of nodes in the *C* which is defined as *k*_*C*_ = ∑_*i* ∈ *C*_ *k*_*i*_, *k*_*i*_ is the degree of node *i*, and *m* is the total number of edges in the network. The value of *Q* ranges from -1 to 1 and the larger value corresponds to better network partition. From the Eq (1), modularity can be regarded as a fixed tradeoff between two terms, i.e. $\sum_{C\in X}\frac{l_{C}}{m}$ and $-\sum_{C\in X}{\left(\frac{k_{C}}{2m}\right)}^{2}$. To maximize the modularity, both terms should be maximized as possible as they can. Maximizing the first term means as many as possible edges should be included in communities, which will lead to larger communities. While maximizing the second term requires the total degree of nodes in communities should be as small as possible, which will lead to smaller communities. These two complementary terms naturally conflict with each other to some extent and reflect the two aspects of a good partition, i.e., densely intra-connected and sparsely interconnected. Thus we select them as two separate objective functions for our algorithm. The first objective function is

$$\begin{array}{c} IntraQ=\sum_{C\in X}\frac{l_{C}}{m}. \end{array}$$ (2)

The value of this term ranges from 0 to 1. Since the range of the second term of modularity is from -1 to 0, we add a constant 1 to regularize it which will not affect the partition results of the network, i.e.,

$$\begin{array}{c} InterQ=1-\sum_{C\in X}{\left(\frac{k_{C}}{2m}\right)}^{2}. \end{array}$$ (3)

Because such two objective functions have opposite propensity to the size and the number of communities, the maximization of them can find community structures at different resolution levels. The MMCD detects community structures by maximizing such two objective functions which is formulated as

$$\begin{array}{c} \max F\left(X\right)=\left\{IntraQ,InterQ\right\},\;\text{subject}\;\text{to}\;\;X=\left(x_{1},x_{2},\ldots ,x_{n}\right)\in \Omega , \end{array}$$ (4)

where *X* is a possible partition in network partition space Ω. Since MMCD integrates a local search procedure into a EA which is proven to solve the above optimization problem successfully, it can solve the above optimization problem by designing an appropriate local search procedure.

## Methods

The MMCD optimizes the formulation Eq (4) by both evolutionary global search and local search in each generation. As our main focus is on local search procedure, we adopt modified framework of NNIA [20] as our multi-objective global search mechanism. In fact, our local search procedure can also be integrated into some other multi-objective optimization evolutionary algorithms, such as NSGA-II and PESA-II.

Some related terms are stated as follows. Feasible solutions of problems are also called individuals or partitions. In each generation of the algorithm, six populations of individuals evolve in turn. They are dominant population *D* which is the set of nondominated individuals, active population *AP* which is the set of individuals selected from dominant population with larger crowding distance, clone population *CL* which stores clones of active individuals, evolutionary search offspring population *EO* which contains the result of evolutionary search, local search offspring population *LO* which contains the result of local search, and combined population *B* which is responsible for elitism. The main procedure of MMCD is given as Algorithm 1 in Table 1.

**Table 1:** Algorithm 1. Main procedure of multi-objective memetic algorithm for community detection.

**Input:** Maximum number of generations *Gmax*, Maximum size of dominant population *SD*, Maximum size of active population *SA*, Size of clone population *SC*, Mutation probability *p*_*m*_, Maximum iterations of local search strategy *MI*, Adjacency matrix of the network *A*.
**Output:** Network partitions at different resolution levels.
1: **Initialization**: Generate an initial population *B*_0_ with size *SD*, set generation count *g* = 0;
2: **Update Dominant Population**: Identify all nondominated individuals in *B*_*g*_. Calculate the crowding-distance values of all nondominated individuals, sort them in descending order of crowding-distance, and choose the first *SD* individuals to form dominant population *D*_*g*_;
3: **Nondominated Neighbor-Based Selection**: If the size of *D*_*g*_ is not larger than *SA*, let *AP*_*g*_ = *D*_*g*_. Otherwise, sort individuals in *D*_*g*_ in descending order of crowding-distance, and select the first *SA* individuals to form *AP*_*g*_. Meanwhile, the original dominant population *D*_*g*_ is set as external population to be applied for elitism;
4: **Proportional Cloning**: Apply proportional cloning to *AP*_*g*_ to obtain the clone population *CL*_*g*_;
5: **Crossover and Mutation**: Perform crossover and mutation operations on *CL*_*g*_ to produce evolutionary search offspring population *EO*_*g*_;
6: **Local Search Procedure**: Perform local search operation with parameter *MI* on external dominant population *D*_*g*_ and *EO*_*g*_ to obtain two local search offspring populations $LO_{g}^{1}$ and $LO_{g}^{2}$, respectively;
7: **Combination**: Combine $LO_{g}^{1}$ and $LO_{g}^{2}$ together to form *B*_*g*+1_. Set *g* = *g*+1;
8: **Termination**: If *g* < *Gmax*, return to step 2. Otherwise, go to step 9;
9: **Model Selection**: Identify all nondominated individuals in *B*_*g*_ to form dominant population *DT*_*g*_ and select solutions in *DT*_*g*_ based on some criteria;
10: **Decoding**: Decode selected solutions into network partitions. Stop.

Bold words denote module names of each steps.

Initialization constructs the initial solution population of the algorithm. Nondominated Neighbor-Based Selection and Proportional Cloning are used to keep nondominated individuals more diverse based on crowding distance [18] to prevent individuals from gathering in some local regions and avoid premature convergence to local optimal solutions. Crossover and Mutation are evolutionary global search operations and Local Search Procedure is local search operation on population. Model Selection select partitions from final approximated Pareto-optimal set. The specific criteria to select solutions at each resolution scale should depend on specific applications. As Initialization are the basis of the algorithm, and evolutionary search operations (Crossover and Mutation) and Local Search Procedure are two search operations responsible for population evolution, we will discuss implementation details of them in the followings.

### Representation and Initialization

**Fig 1:**
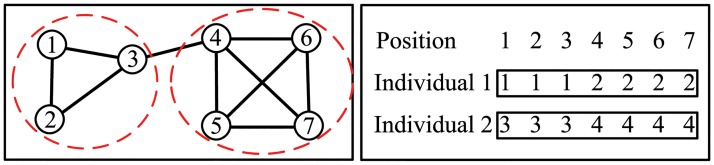
Illustration of group based representation. Left, a network with a community structure. Right, two possible representations corresponding to the community structure.

Locus-based adjacency representation [27] and group based representation [28] are two common used representation strategies for community partitions in evolutionary algorithms. In this study, we use the latter one as it is more straightforward and can be conveniently handled in local search procedure. Each individual *X*_*p*_ in the population is encoded as $X_{p}=\{x_{p}^{1},x_{p}^{2},\ldots ,x_{p}^{n}\}$, where *n* is the number of nodes in the network and $x_{p}^{i}$ denotes the community identifier (also named as community label) of node *i* in partition *X*_*p*_. As at most there are *n* communities in the network with *n* nodes, the value of $x_{p}^{i}$ can be chosen from any integer in the range of 1 to *n*. In the decoding step, the nodes with the same community label are grouped into one community. If there are *k* different community labels in an individual at the end of algorithm, the community structure corresponding to this individual will automatically have *k* communities. One prominent property of this algorithm is that the number of community *k* is unnecessary to set in advance. It is worth noting that there are multiple representations corresponding to one community structure. An illustration of this representation strategy for a toy network is given in Fig 1. The network with 7 nodes is partitioned into two communities. Two possible representations corresponding to the community structure are *X*_1_ = {1,1,1,2,2,2,2} and *X*_2_ = {3,3,3,4,4,4,4}.

The population initialization procedure is given as Algorithm 2 in Table 2. Initially, each node is put in a different community for all individuals in the initial population. Assume each individual has the same community label assignment (i.e. *X*_*p*_ = {1,2,…, *n*},∀*p* = 1,2,…, *SI*). Then a simple heuristic process is employed which is similar to that in [28]. For each individual, randomly select a portion of nodes (e.g., *α* ⋅ *n* nodes, *α* is a parameter and set as *α* = 0.3 for experiments in this paper) and assign their community labels to all of their neighbors, respectively. This heuristic process enhances the diversity and provides the better quality of the initial solutions, and has a low computational complexity.

**Table 2:** Algorithm 2. Population initialization procedure.

**Input:** Initial Population size *SI*.
**Output:** Initial Population *B*_0_.
1: Generate *SI* individuals, $X_{p}=\{x_{p}^{1},x_{p}^{2},\ldots ,x_{p}^{n}\},1\leq p\leq SI,x_{p}^{i}\;\leftarrow \;i,1\leq i\leq n$;
2: *B*_0_ ← ∅
3: **for** each individual *X*_*p*_ **do**
4: Generate a random sequence, i.e. {*a*_1_, *a*_2_,…, *a*_*n*_};
5: **for** each of first *α* ⋅ *n* nodes $x_{p}^{a_{i}}$ in this sequence **do**
6: $x_{p}^{j}\leftarrow x_{p}^{a_{i}},\forall j\in \{j|A_{a_{i}j}=1\}$;
7: **end for**
8: *B*_0_ ← *B*_0_∪{*X*_*p*_};
9: **end for**

### Crossover and Mutation

**Fig 2:**
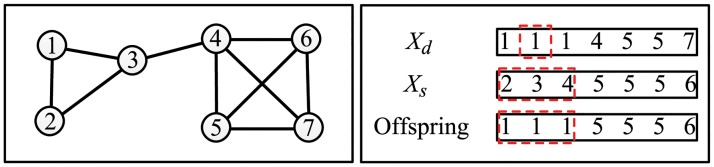
Illustration of one-way crossover procedure. Left, a toy network with 7 nodes; Right, node 2 is randomly chosen and the set of nodes with the same label as node 2 is {1,2,3} in *X*_*s*_.

Crossover is one of the evolutionary search operations in NNIA [20]. According to NNIA, the crossover operation *O* ^*R*^ on the clone population *CL* can be defined as

$$\begin{array}{ccc} O^{R}\left(CL\right) & = & O^{R}\left(c_{1}\right)+O^{R}\left(c_{2}\right)+\cdots +O^{R}\left(c_{SC}\right)\\ & = & crossover\left(c_{1},AP\right)+crossover\left(c_{2},AP\right)+\cdots +crossover\left(c_{SC},AP\right), \end{array}$$

where *crossover*(*c*_*i*_, *AP*) represents crossover operator on clone *c*_*i*_ and an active individual randomly selected from population *AP*. In order to maintain population size, *crossover*(*c*_*i*_, *AP*) needs to return one offspring to replace *c*_*i*_. Two-point crossover [13] and uniform crossover [21] are two commonly used crossover operations, but they are not appropriate here because of a property of group based representation strategy. In this representation strategy, the same community label in different individuals may represents different communities, so community labels can’t be simply exchanged between different individuals. In this study, we employ one-way crossover operation [28]. The crossover procedure is given as Algorithm 3 in Table 3. Assuming *X*_*p*_ and *X*_*q*_ are two crossover operation parents. Randomly select one of them as source individual *X*_*s*_ and the other one as destination individual *X*_*d*_. Then, randomly choose a node *i* whose community labels in *X*_*s*_ is $x_{s}^{i}$. Identify the set of nodes with the same community label as $x_{s}^{i}$ in *X*_*s*_ and replace the labels of this set of nodes in *X*_*d*_ with $x_{s}^{i}$, i.e. $x_{d}^{j}\leftarrow x_{s}^{i},\forall \;j\in \{j|x_{s}^{j}=x_{s}^{i}\}$. This procedure is illustrated in Fig 2. Assuming the source individual is *X*_*s*_ = {1,1,1,4,5,5,7} and the destination individual is *X*_*d*_ = {2,3,4,5,5,5,6}. Node 2 is randomly chosen and its community label in *X*_*s*_ is 1. The set of nodes {1,2,3} has the same community label as node 2 in *X*_*s*_. Replacing labels of all nodes in this set with label 1 in *X*_*d*_ to produce the offspring.

**Table 3:** Algorithm 3. One-way crossover procedure.

**Input:** Two individuals *X*_*p*_ and *X*_*q*_.
**Output:** Offspring of crossover operation *X*_*o*_.
1: Generate a random value *r* ∈ [0, 1];
2: Randomly choose a node *v*_*i*_;
3: **if** *r* < 0.5 **then**
4: Identify the set of nodes *p* ^*i*^ with the same community label as $x_{p}^{i}$ in *X*_*p*_, i.e. $p^{i}\leftarrow \{j|x_{p}^{j}=x_{p}^{i}\}$;
5: $x_{q}^{j}\leftarrow x_{p}^{i},\forall j\in p^{i}$;
6: *X*_*o*_ ← *X*_*q*_;
7: **else**
8: Identify the set of nodes *q* ^*i*^ with the same community label as $x_{q}^{i}$ in *X*_*q*_, i.e. $q^{i}\leftarrow \{j|x_{q}^{j}=x_{q}^{i}\}$;
9: $x_{p}^{j}\leftarrow x_{q}^{i},\forall j\in q^{i}$;
10: *X*_*o*_ ← *X*_*p*_;
11: **end if**

Mutation is the other evolutionary search operation. A neighbor-based mutation operation which reduces the useless search space by taking into account the effective connections among nodes is used [12]. In this operation, the reassigned labels of mutated nodes are restricted to those of their neighbors. For each node *i* of each individual *X*_*p*_ generated by crossover operation, generating a random value *r* ∈ [0, 1]. If *r* is smaller than the mutation probability *p*_*m*_ (*p*_*m*_ is set as 0.01 for MMCD in this paper), the community label of this node is replaced with one of its neighbors’ label, i.e. $x_{p}^{i}\leftarrow x_{p}^{j},\exists \;j\in \{j|A_{ij}=1\}$.

### Local Search Procedure

Different from memetic algorithms for single objective community detection problems, the cases of multi-objective face some new problems. We summarize these problems into three issues, i.e., determining initial individuals, defining fitness function and designing local search strategy.

#### Initial individuals

In each generation, local search procedure is applied to “good” individuals in two populations to obtain better individuals. One is evolutionary search offspring population *EO* generated after mutation operation and the other one is external dominant population *D* used for the purpose of elitism. Since non-dominated individuals are “good” individuals in multi-objective cases, initial individuals for local search procedure are nondominated solutions in population *EO* and solutions in population *D*.

#### Fitness function

**Fig 3:**
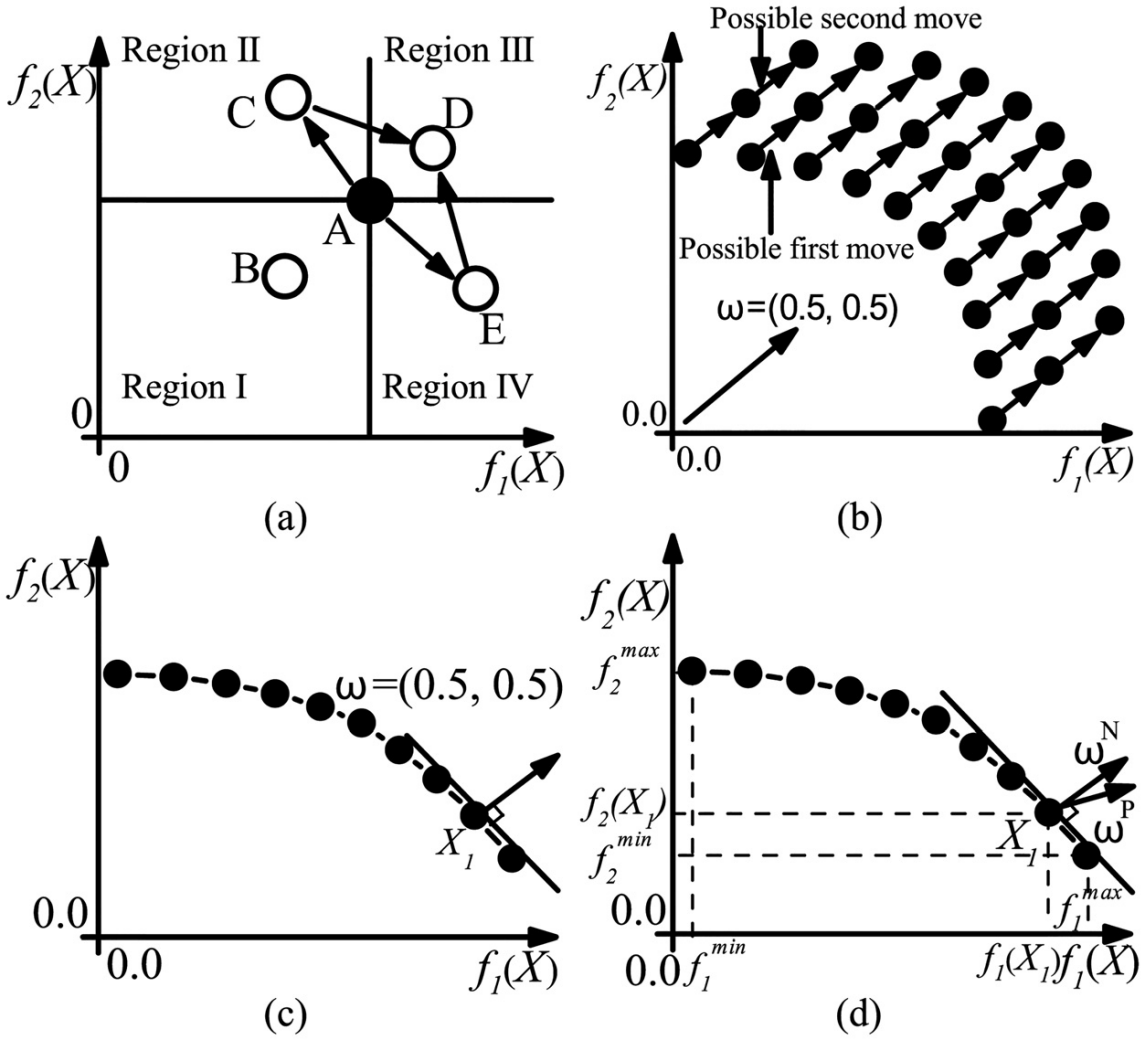
Illustration of possible designs of fitness evaluation method for local search procedure. (a) Region I-IV are four regions divided with respect to node A. Individuals in region I dominate A and in region I,II,IV are not dominated by A. Region I is too small to search, while individuals in region II and IV may move to Region III which are dominated by A after several generations; (b) When constant weight vector *ω* = (0.5,0.5) is applied, individual population will suffer from diversity problem after several searches; (c) Assuming weight vector *ω* = (0.5,0.5) selects *X*_1_ as initial individual according to random weight vector scheme, then the probability to select right side individual is much higher then select left side as there is only one individual on the right side of *X*_1_; (d) Pseudoweight vector *ω* ^*P*^ deviates from normal line vector *ω* ^*N*^.

There are two objective functions in MMCD, while local search procedure needs a single fitness function to evaluate the neighbors of an initial individual to decide which neighbor can replace the initial one as a better solution. Dominance relation which is used in multi-objective EAs can be simply used as a fitness evaluation method by which an initial individual can be replaced with one that dominates it or the one that is not dominated by it. As is illustrated in Fig 3(a), the movable area of the former fitness evaluation rule is too small to find satisfied neighbors, while the area is too huge for the latter one which allows to select individuals in dominated regions after several searches. Besides dominance relation, scalarizing function is widely used as the fitness function for local search procedure in MOPs [29, 30]. Scalarizing fitness function based on two objective funtions can be expressed as

$$\begin{array}{c} S\left(X\right)=\omega_{1}f_{1}\left(X\right)+\omega_{2}f_{2}\left(X\right), \end{array}$$

where *f*_1_(*X*) = *IntraQ*, *f*_2_(*X*) = *InterQ* for our method, and *ω*_1_ and *ω*_2_ are nonnegative weights, which satisfy the following constraints

$$\begin{array}{c} \omega_{i}\geq 0,\;i=1,2\\ \omega_{1}+\omega_{2}=1. \end{array}$$

Determining the appropriate weights is crucial for effective and efficient local search procedure. As is illustrated in Fig 3(b), if a constant weight vector ***ω*** = (*ω*_1_, *ω*_2_) is applied, all initial individuals will perform local search in one fixed direction which will result in serious diversity problem for evolutionary search in the next generation. To maintain population diversity, the weight vector (i.e. local search direction in objective space) should depend on the location of each initial individual. A random weight vector scheme is proposed in [29] which first randomly specifies a weight vector and then select a solution with maximal fitness function value in that direction as initial individual for this weight vector. However, as is illustrated in Fig 3(c), this scheme has a bias selection problem that individuals in a small region may be selected with much higher probability, when the value range distributions of initial individuals in two objective functions vary a lot. What’s more, some individuals may be selected more than one time while some others may not be selected any more. Pseudoweight vector [31] is another widely used weight vector for scalarizing fitness function and it is defined as

$$\begin{array}{c} \omega_{i}^{P}\left(X\right)=\frac{\left(f_{i}\left(X\right)-f_{i}^{min}\right)/\left(f_{i}^{max}-f_{i}^{min}\right)}{\sum_{j=1}^{M}\left(f_{j}\left(X\right)-f_{j}^{min}\right)/\left(f_{j}^{max}-f_{j}^{min}\right)},\;i=1,2,\ldots ,M, \end{array}$$

where *M* is the number of objective functions which is 2 in MMCD algorithm. Pseudoweight vector roughly denotes the priorities of different objective functions at each individual. As is illustrated in Fig 3(d), sometimes pseudoweight vector may obviously deviate from the normal line of nondominated front. Nondominated front here is defined as the images of all nondominated solutions in objective space and normal line is the line perpendicular to the smooth line in nondominated front. In fact, the nondominated front in objective space can be regarded as a generalized isoline, because points in this front are not better or worse than each other. Since the gradient direction of an isoline is its normal line direction, the ideal direction of local search should also be the direction of normal line of the nondominated front.

**Fig 4:**
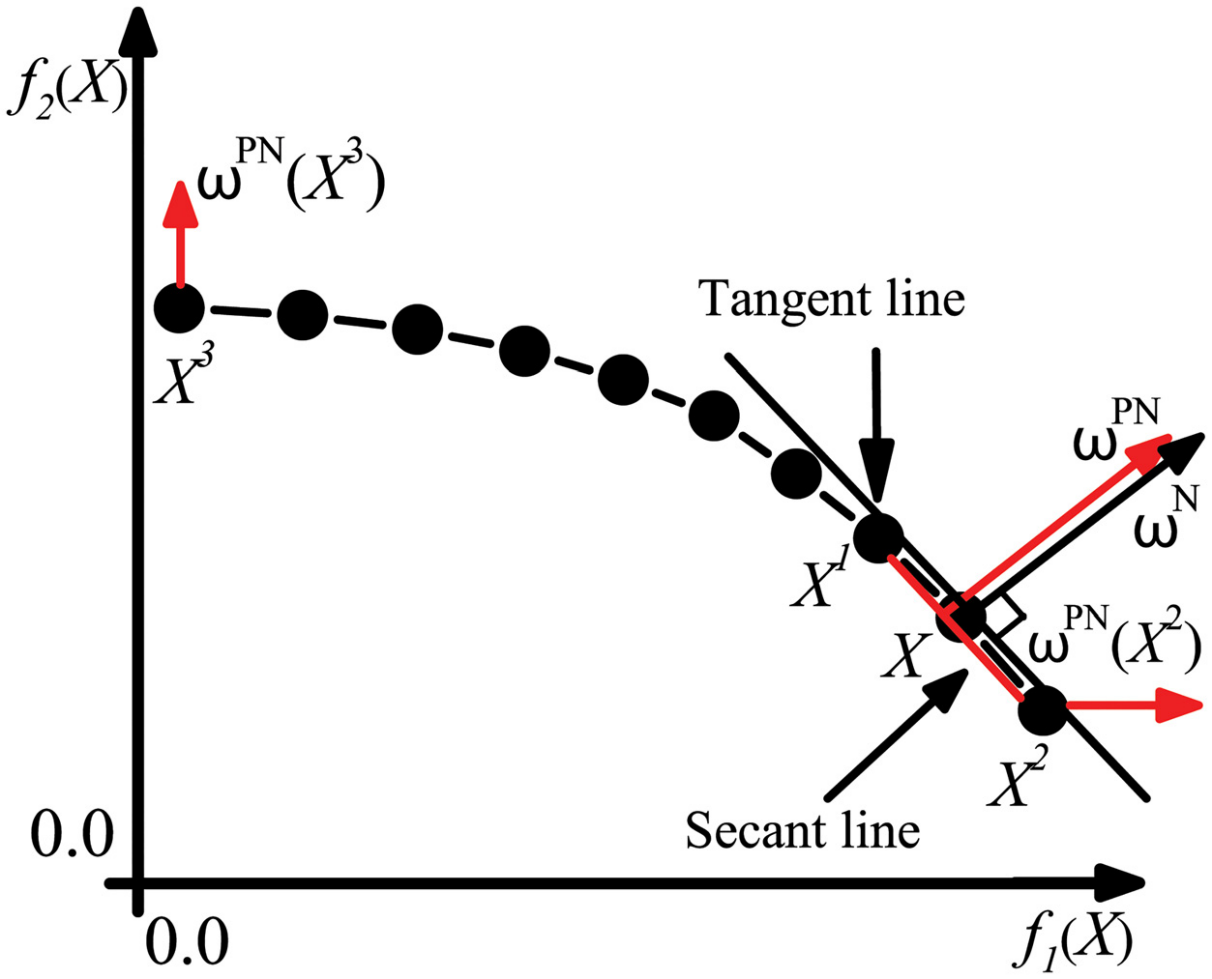
Illustration of pseudonormal vector. *ω* ^*N*^ is the vector of normal line, *ω* ^*PN*^ is the pseudonormal vector.

As it is known to all, normal line is perpendicular to tangent line in two-dimensional space. However, the smooth line in nondominated front is not an inerratic curve and its formula is usually unknown, so the tangent line can’t be derived easily. Thus, we propose the pseudonormal vector which approximates the normal line vector for our two objective optimization problem. In fact, tangent line derives from secant line and it is the limiting case of the secant line, so we can apply secant line to approximate tangent line and set pseudonormal line perpendicular to such approximated tangent line. As is illustrated in Fig 4, for an arbitrary individual *X* which is not one of the boundary individuals in the nondominated front, the slope of tangent line at the location of *X* is similar to the slope of secant line connecting two individuals *X* ^1^ and *X* ^2^ that are closest to *X* on either side. The “closest to *X* on either side” here means closest to *X* on either side along either objective function. Actually, two individuals in nondominated front closest to *X* on either side along one objective function are also closest to *X* on either side along the other one. To prove this property, we first give another property of nondominated front in two-dimensional objective space.

**Property 1 (Reversed order property)** *In two-dimensional objective space, the ascending order of individuals in nondominated front along one objective function value is the descending order along the other objective function value.*

*Proof.* Individuals in nondominated front do not dominate each other, so one individual greater than another one along one objective function value must be smaller than that one along the other objective function value in nondominated front of two-dimensional objective space. The property is proved by iterating such relation to all pairs of individuals in nondominated front.

**Property 2 (Closest relation property)** *In two-dimensional objective space, for an arbitrary individual X which is not a boundary individual in nondominated front, two individuals in nondominated front closest to X on either side along one objective function are also closest to X on either side along the other one.*

*Proof.* Assuming *X* ^1^ and *X* ^2^ are two individuals closest to *X* along objective function *f*_1_ and the relation of inequality is *f*_1_(*X* ^1^) < *f*_1_(*X*) < *f*_1_(*X* ^2^). According to Property 1, we have *f*_2_(*X* ^1^) > *f*_2_(*X*) > *f*_2_(*X* ^2^). If two other individuals *X* ^3^ and *X* ^4^ are closest to *X* along objective function *f*_2_ and the relation of inequality is *f*_2_(*X* ^3^) > *f*_2_(*X*) > *f*_2_(*X* ^4^), then we have the relation of inequality that *f*_2_(*X* ^1^) > *f*_2_(*X* ^3^) > *f*_2_(*X*) > *f*_2_(*X* ^4^) > *f*_2_(*X* ^2^). However, according to Property 1, it is easy to obtain that *f*_1_(*X* ^1^) < *f*_1_(*X* ^3^) < *f*_1_(*X*) < *f*_1_(*X* ^4^) < *f*_1_(*X* ^2^) which is contradictory to the assumption that *X* ^1^ and *X* ^2^ are two individuals closest to X along objective function *f*_1_. Thus, the property is proved.

The secant line connecting two individuals *X* ^1^ and *X* ^2^ is

$$\begin{array}{c} s=(f_{1}\left(X^{2}\right)-f_{1}\left(X^{1}\right),f_{2}\left(X^{2}\right)-f_{2}\left(X^{1}\right)). \end{array}$$

Then the pseudonormal vector of *X* is set perpendicular to such secant and defined as

$$\begin{array}{c} \omega^{PN}\left(X\right)=\left(\frac{f_{2}\left(X^{1}\right)-f_{2}\left(X^{2}\right)}{\pi },\frac{f_{1}\left(X^{2}\right)-f_{1}\left(X^{1}\right)}{\pi }\right), \end{array}$$ (5)

where *π* is the normalization factor, i.e. *π* = *f*_2_(*X* ^1^) − *f*_2_(*X* ^2^)+*f*_1_(*X* ^2^) − *f*_1_(*X* ^1^). For boundary individuals (i.e. *X* ^2^ and *X* ^3^ in Fig 4) who are in the boundary of nondominated front, only one side of them has closest individuals, so their pseudonormal vectors cannot be calculated by formula Eq (5). In order to maintain diversity and obtain solutions which are optimal on either objective function, the pseudonormal vectors of boundary individuals are set parallel to coordinate axes, i.e. *ω* ^*PN*^(*X* ^2^) = (1,0) and *ω* ^*PN*^(*X* ^3^) = (0,1) in Fig 4. Note that the local search direction specified by the weight vector *ω* in the objective space is a totally different concept from that in the decision space. The search direction here is used to judge the quality of individuals and guide the local search rather than the exact direction which individuals move along.

#### Local search strategy

By using the pseudonormal vector *ω* ^*PN*^, the scalarizing fitness function for local search procedure in MMCD algorithm is

$$\begin{array}{c} S\left(X\right)=\omega_{1}^{PN}IntraQ+\omega_{2}^{PN}InterQ. \end{array}$$ (6)

By substituting *IntraQ* and *InterQ* with formula Eq (2) and Eq (3), respectively, the fitness function can be written as

$$\begin{array}{ccc} S(X) & = & \sum_{C\in P}S\left(C\right)+\omega_{2}^{PN},\\ S(C) & = & \omega_{1}^{PN}\frac{l_{C}}{m}-\omega_{2}^{PN}{\left(\frac{k_{C}}{2m}\right)}^{2}. \end{array}$$ (7)

where the meanings of parameters are the same as those in formula Eq (2) and Eq (3) and *S*(*C*) is the contribution of community *C* to fitness function *S*(*X*). For a conventional local search procedure, the neighborhood of an initial individual is first defined. Then the fitness values of all neighbors are calculated and the neighbor with maximal fitness value is selected to replace the current individual. However, calculating the fitness value from scratch for all neighbors is one of the most time consuming operations in the algorithm. So inspired by LPAm [24] which local optimizes modularity, we apply an efficient local search strategy here which only calculates the increments of fitness value based on network structure information.

Firstly, we define the neighborhood of a network partition (i.e. individual or solution). Intuitively, a neighbor partition should be close to or similar to itself. Some definitions are given as follows.

**Definition 1 (Local neighborhood)** *For a network partition X*_*p*_ *, the local neighborhood N*_*i*_ *(X*_*p*_ *) respect to a node v*_*i*_ *is defined as the set of partitions formed by moving node v*_*i*_ *to one of its adjacent communities, i.e.,*

$$\begin{array}{c} N_{i}\left(X_{p}\right)=\{X|\{\begin{array}{ccc} x^{j}=x_{p}^{j}, & \mathrm{if} & j\neq i,\\ x^{j}=x_{p}^{AC_{i}}, & \mathrm{if} & j=i. \end{array}\}, \end{array}$$

*where* $x_{p}^{{AC}_{i}}$ *is the community label of community AC*_*i*_ *in partition X*_*p*_ *, AC*_*i*_ *is an adjacent community of v*_*i*_ *which is defined as*

$$\begin{array}{c} AC_{i}\in \{C_{j}|v_{j}\in N\left(v_{i}\right)\}, \end{array}$$

*where C*_*j*_ *is the community which node v*_*j*_ *belongs to and N* *(v*_*i*_ *) is the set of neighbors of node v*_*i*_ *.*

*N(v*_*i*_ *) includes v*_*i*_ *itself here, thus community of node itself is also regarded as its adjacent community and it is easy to derive that X*_*p*_ *∈ N*_*i*_ *(X*_*p*_ *).*

**Definition 2 (Neighborhood)** *For a network partition X*_*p*_ *, the neighborhood N* *(X*_*p*_ *) is defined as the union of local neighborhood respect to all nodes, i.e.,*

$$\begin{array}{c} N\left(X_{p}\right)=\cup_{i=1}^{n}N_{i}\left(X_{p}\right). \end{array}$$

Next, fitness value increments of partitions in local neighborhood *N*_*i*_(*X*_*p*_) relative to partition *X*_*p*_ are calculated based on the network structure information. The node *v*_*i*_ is set as an isolated community by regarding community of itself as its adjacent community. The fitness increment of a local neighbor corresponding to the adjacent community *AC*_*i*_ in *N*_*i*_(*X*_*p*_) relative to *X*_*p*_ is calculated as

$$\begin{array}{c} \Delta S_{i}\left(X_{p}\right)=S\left(AC_{i}+v_{i}\right)-S\left(AC_{i}\right)-S\left(\left\{v_{i}\right\}\right), \end{array}$$ (8)

where *AC*_*i*_+*v*_*i*_ denotes the community combining *AC*_*i*_ with *v*_*i*_ and {*v*_*i*_} denotes the isolated community which only contains *v*_*i*_. After calculating all increments corresponding to all adjacent communities *AC*_*i*_ of *v*_*i*_, the one with maximal increment is chosen as *v*_*i*_’s new community. If the new community is the same as the original one of *v*_*i*_, it means *v*_*i*_ is already in the local best community in partition *X*_*p*_ and *X*_*p*_ is a local maximal solution in local neighborhood *N*_*i*_(*X*_*p*_). In order to show how to calculate Δ*S*_*i*_(*X*_*p*_), the formula Eq (8) is expanded as

$$\begin{array}{ccc} \Delta S_{i}\left(X_{p}\right) & = & \left[\omega_{1}^{PN}\frac{l_{AC_{i}+v_{i}}}{m}-\omega_{2}^{PN}{\left(\frac{k_{AC_{i}+v_{i}}}{2m}\right)}^{2}\right]\\ & & -[\omega_{1}^{PN}\frac{l_{AC_{i}}}{m}-\omega_{2}^{PN}{\left(\frac{k_{AC_{i}}}{2m}\right)}^{2}]\\ & & -[-\omega_{2}^{PN}{\left(\frac{k_{i}}{2m}\right)}^{2}]\\ & = & \omega_{1}^{PN}\frac{l_{AC_{i},v_{i}}}{m}-\omega_{2}^{PN}\frac{k_{AC_{i}}k_{i}}{2m^{2}}, \end{array}$$ (9)

where *l*_*AC*_*i*_+*v*_*i*__ and *l*_*AC*_*i*__ are the number of edges in the community *AC*_*i*_+*v*_*i*_ and *AC*_*i*_, respectively. *l*_*AC*_*i*_, *v*_*i*__ is the number of edges between node *v*_*i*_ and the nodes in *AC*_*i*_. *k*_*AC*_*i*_+*v*_*i*__ and *k*_*AC*_*i*__ are the total degree of nodes in the *AC*_*i*_+*v*_*i*_ and *AC*_*i*_ respectively and *k*_*i*_ is the degree of node *v*_*i*_. As mentioned above, the community labels of node *v*_*i*_ and community *AC*_*i*_ in *X*_*p*_ are $x_{p}^{i}$ and $x_{p}^{AC_{i}}$, respectively. The formula Eq (9) can be rewritten in the form of adjacency matrix and community labels as

$$\begin{array}{c} \Delta S_{i}\left(X_{p}\right)=\frac{1}{m}\sum_{j\neq i}(\omega_{1}^{PN}A_{ij}-\frac{\omega_{2}^{PN}k_{i}k_{j}}{2m})\delta \left(x_{p}^{j},x_{p}^{AC_{i}}\right), \end{array}$$ (10)

where *δ* is the Kronecker delta function. Finally the new community label $x_{p}^{i^{\prime }}$ of node *i* should be updated to $x_{p}^{AC_{i}}$ which maximizes the formula (10), i.e.

$$\begin{array}{c} x_{p}^{i^{\prime }}=\arg\max_{x_{p}^{AC_{i}}}\sum_{j\neq i}(\omega_{1}^{PN}A_{ij}-\frac{\omega_{2}^{PN}k_{i}k_{j}}{2m})\delta \left(x_{p}^{j},x_{p}^{AC_{i}}\right). \end{array}$$ (11)

This fitness function specialized label update rule can be efficiently implemented by an algorithm similar to label propagation algorithm [23]. The local search procedure is illustrated in Algorithm 4 in Table 4. The local optimum around *X*_*p*_ in its neighborhood is obtained by getting local optimism in its local neighborhoods respect to all different nodes in a random sequence. The local search procedure will be terminated when a predefined maximum iterations *MI* is reached or the community label of each node is unchanged.

**Table 4:** Algorithm 4. Local Search Procedure.

**Input:** Individual population *P*, Adjacency matrix *A*, Maximum iterations *MI*.
**Output:** Offspring population of local search operation *OP*.
1: Select all nondominated individuals in *P* to form population *DP*;
2: **for** each individual *X* in *DP* **do**
3: Calculate pseudonormal vector *ω* ^*PN*^(*X*);
4: *count* ← 0; *ischanged* ← 1; *OP* ← ∅;
5: **while** *count* < *MI*∧*ischanged* == 1 **do**
6: *ischanged* ← 0; *count* ← *count*+1;
7: Generate a random sequence, i.e. {*a*_1_, *a*_2_,…, *a*_*n*_};
8: **for** each node *v*_*a*_*i*__ in random sequence **do**
9: Update label $x_{p}^{a_{i}}$ to $x_{p}^{{a_{i}}^{\prime }}$ with fitness function specialized label propagation rule (11);
10: **if** $x_{p}^{a_{i}}\neq x_{p}^{{a_{i}}^{\prime }}$ **then**
11: *ischanged* ← 1;
12: **end if**
13: **end for**
14: **end while**
15: *OP* ← *OP*∪{*X*};
16: **end for**

### Analysis of Computational Complexity

In this section, the computational complexity of the proposed algorithm MMCD is analyzed. Given a network with *n* nodes and *m* edges and assuming that the maximum size of the dominant population and active population is *SD* and *SA*, respectively, the size of clone population is *SC*, the time complexity of one generation for the algorithm can be calculated as follows.

The time complexity for initialization procedure is *O*(*SD* ⋅ *n*). The calculation of two objective functions needs *O*(*m*) time. At most *O*(*SD*+*SC*) calculations of objective functions and *O*((*SD*+*SC*)^2^) comparisons are required to identify nondominated individuals in combined population. The worst time complexity for sorting individuals by crowding distance is *O*((*SD*+*SC*)log(*SD*+*SC*)). According to analysis in [20], proportional cloning requires *O*(*SC*) time. The crossover and mutation operations in this paper cost *O*(*SC* ⋅ *n*). So the total time complexity for multi-objective evolutionary search procedure in one generation is *O*((*SD*+*SC*) ⋅ *m*+(*SD*+*SC*)^2^+(*SD*+*SC*)log(*SD*+*SC*)+*SC*+*SC* ⋅ *n*). As for local search procedure, identifying nondominated individuals requires *O*(*SC* ^2^) comparisons and *O*(*SC*) calculations of objective functions. For each individual, calculating pseudonormal vector requires constant time and the time complexity for label propagation procedure is at most *O*(*MI* ⋅ *m*) according to analysis in [24], where *MI* is the maximum iterations for label propagation. Thus, the total time complexity for local search procedure in one generation is at most *O*(*SC* ⋅ *m*+*SC* ^2^+*SC* ⋅ *MI* ⋅ *m*). Based on the analysis above, according to the operational rules of the symbol *O*, the overall time complexity of MMCD algorithm with *g* generations is *O*(*g* ⋅ ((*SD*+*SC*) ⋅ *m*+(*SD*+*SC*)^2^+*SC* ⋅ *MI* ⋅ *m*)). Maximum iterations *MI* is usually a very small constant, as 95% nodes or more usually can be partitioned correctly by only 5 iterations in LPA [23]. For large network, *m* will be much larger than *SD*+*SC*, so the time complexity can be further simplified to *O*(*g* ⋅ (*SD*+*SC*) ⋅ *m*).

## Experimental Results

In this section, we will study the MMCD through experiments on artificial and real-world networks from three aspects. The first experiments will discuss the influence of parameters in MMCD and validate the advantages of local search procedure in MMCD. Then MMCD will be compared with a variety of classic community detection algorithms on artificial and real-world networks to illustrate the performance on finding community structures. Finally, we will apply the MMCD to detect community structures at different resolution levels on artificial and real-world networks. The MMCD algorithm has been written in MATLAB. Unless stated, all the non-deterministic algorithms have been independently run 10 times on each datasets. The experiments are carried out on a 2.80GHz and 3.00G RAM computer running Windows 7.

Evaluating the performance of a community detection algorithm needs to define a criterion to measure the quality of the obtained partition [14]. Modularity (*Q*) which is used as an objective function for a variety of community detection methods is a natural quality function whose higher value indicates stronger community structure. In fact, maximizing two objective functions of MMCD is also an indirect way to maximize the modularity. On the other hand, if some networks have ground-truth partitions or true partitions, the similarity between true partitions and partitions obtained by algorithms can be calculated to indicate the partition quality. Normalized Mutual Information (*NMI*) proposed by Danon et al. [32] is such a similarity measure. The value of Normalized Mutual Information *NMI*(*P*_1_, *P*_2_) of two partitions *P*_1_ and *P*_2_ is between 0 and 1 with higher value indicating more similar to each other. *NMI* is independent with two objective functions of MMCD because it is based on true partitions of network. In this paper, higher values of both evaluation criteria are regarded as indications of better partitions.

Since multi-objective community detection algorithms can obtain multiple partitions in one run, we use modularity as model selection criterion when it is adopted as quality metric, and use *NMI* as model selection criterion when *NMI* is adopted as quality metric for all multi-objective community detection algorithms in this paper.

### Influences of Parameters and Local Search Procedure

We will first discuss the influence of parameters of MMCD and validate the advantages of local search procedure in MMCD. In order to analyze methods in details, artificial benchmark network named GN benchmark proposed by Girvan and Newman [1] is adopted. The network consists of 128 nodes which are divided into four communities with 32 nodes each. Edges are randomly placed between nodes independently. Each node has an average degree of 16 and shares a fraction 1 − *μ* of edges with the nodes of its community, and a fraction *μ* of edges with the rest of the network. *μ* is called the mixing parameter. When *μ* < 0.5, the average number of neighbors of each node inside its community is larger than that of neighbors belonging to the rest communities, in which case the network has strong community structure. With the increase of *μ*, the community structure will become vaguer and harder to detect by algorithms. 13 different benchmark networks with values of *μ* ranging from 0.0 to 0.6 with step size 0.05 are generated as GN benchmark datasets in our experiments. Experiments about influences of parameters and local search procedure are carried out on GN artificial datasets.

**Fig 5:**
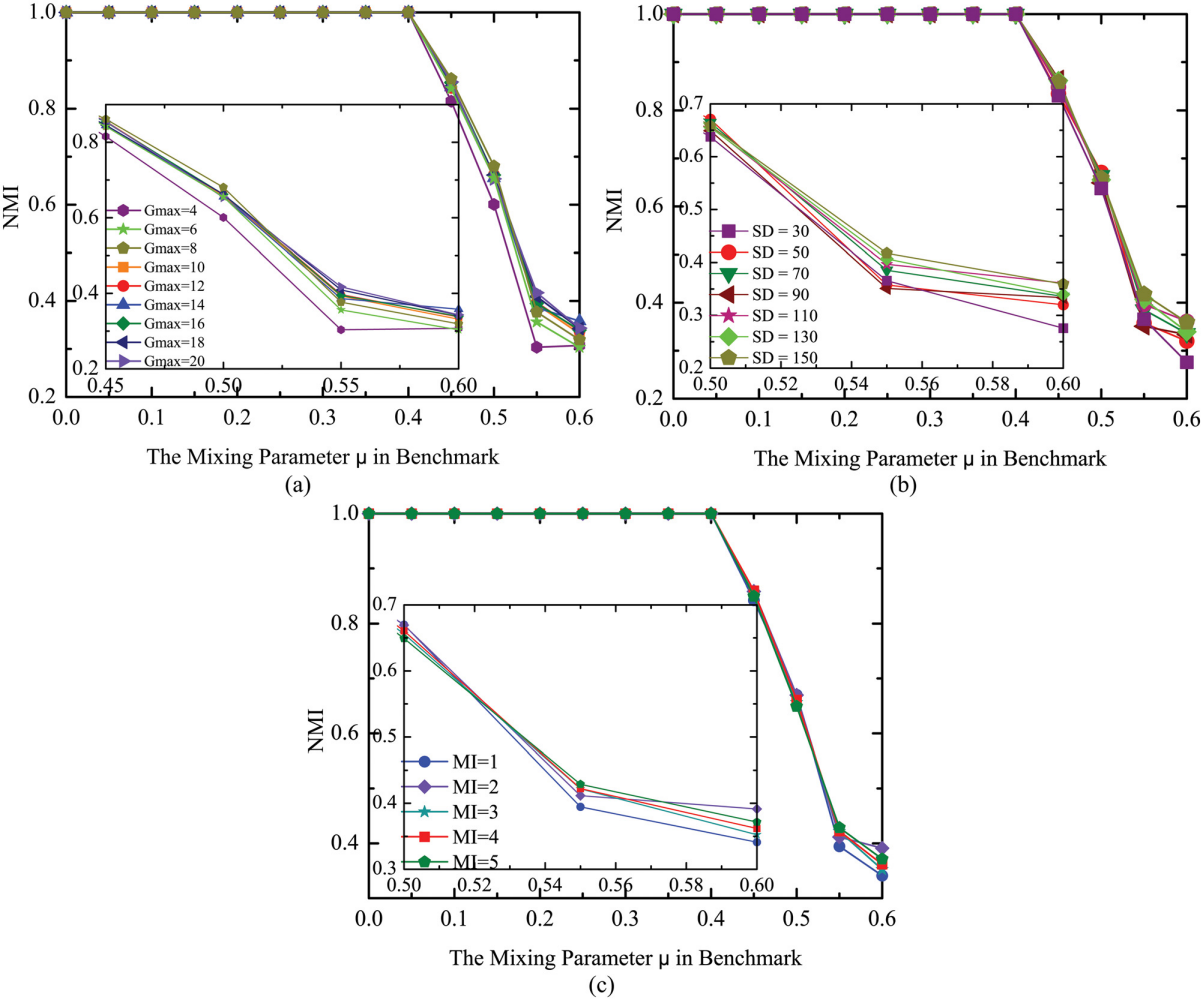
Best *NMI* values averaged over 10 runs for MMCD. (a) Different number of generations; (b) Different maximum sizes of dominant population; (c) Different maximum iteration numbers.

In MMCD, the parameter *Gmax* is used to determine the maximum number of generations. The parameter *SD* indicates the maximum size of dominant population. To simplify the discussion, the maximum size of active population *SA* and size of clone population *SC* are set as *SA* = *SD*/5 and *SC* = *SD* for all experiments in this paper. The parameter *MI* denotes maximum iterations in local search procedure. Firstly, to test the influence of parameter *Gmax* on performance of MMCD, the other two parameters are set as *SD* = 110 and *MI* = 1. After running the algorithm 10 times with different *Gmax*, the average results are shown in Fig 5(a). The results at the range of *μ* ∈ [0.45,0.6] are amplified inside the figure. Since the size of benchmark network is not large, the difference among the results is modest. However, it can be still observed that when mixing parameter *μ* is above 0.4, the results with larger *Gmax* are generally better than those with smaller *Gmax* except for a few cases such as *Gmax* = 8 at *μ* = 0.45 and at *μ* = 0.50. Since MMCD is a non-deterministic algorithm, these exceptions may be due to the nondeterminacy of the algorithm. The difference between results with larger *Gmax* gets smaller. This phenomenon agrees with the general convergence rule that when objective function values are closer to their best values, they will converge more slowly or even stop converging. What’s more, we can see that our algorithm converges rather fast. In order to get the balance between performance quality and running time, the *Gmax* is set to 10 for MMCD on GN benchmark datasets.

Secondly, the influence of parameter *SD* is studied by letting it change from 30 to 150 with interval 20 and fixing *Gmax* = 10, *MI* = 1. The whole average results and the part amplified results at the range of *μ* ∈ [0.5,0.6] are shown in Fig 5(b). It is shown that when *μ* ≤ 0.4, all of them detect the true community structure. With the increase of *μ*, algorithms with larger *SD* generally perform slightly better except for a few cases such as results of *SD* = 50 at *μ* = 0.5 and of *SD* = 90 at *μ* = 0.55. In fact, larger population has bigger probability to include the better individual, but leads to longer running time at the same time. We set *SD* = 110 for MMCD on GN benchmark datasets as it can get ideal results within reasonable running time.

Finally, the influence of maximum iterations of local search procedure *MI* is tested by fixing *SD* = 110 and *Gmax* = 10. The whole results and the part amplified results are shown in Fig 5(c). The results of different *MI* are almost the same even when *μ* > 0.4. The tiny differences are disordered which may result from nondeterminacy of MMCD. So the influence of *MI* on MMCD is negligible and it is set as 1 for all experiments in this paper.

**Fig 6:**
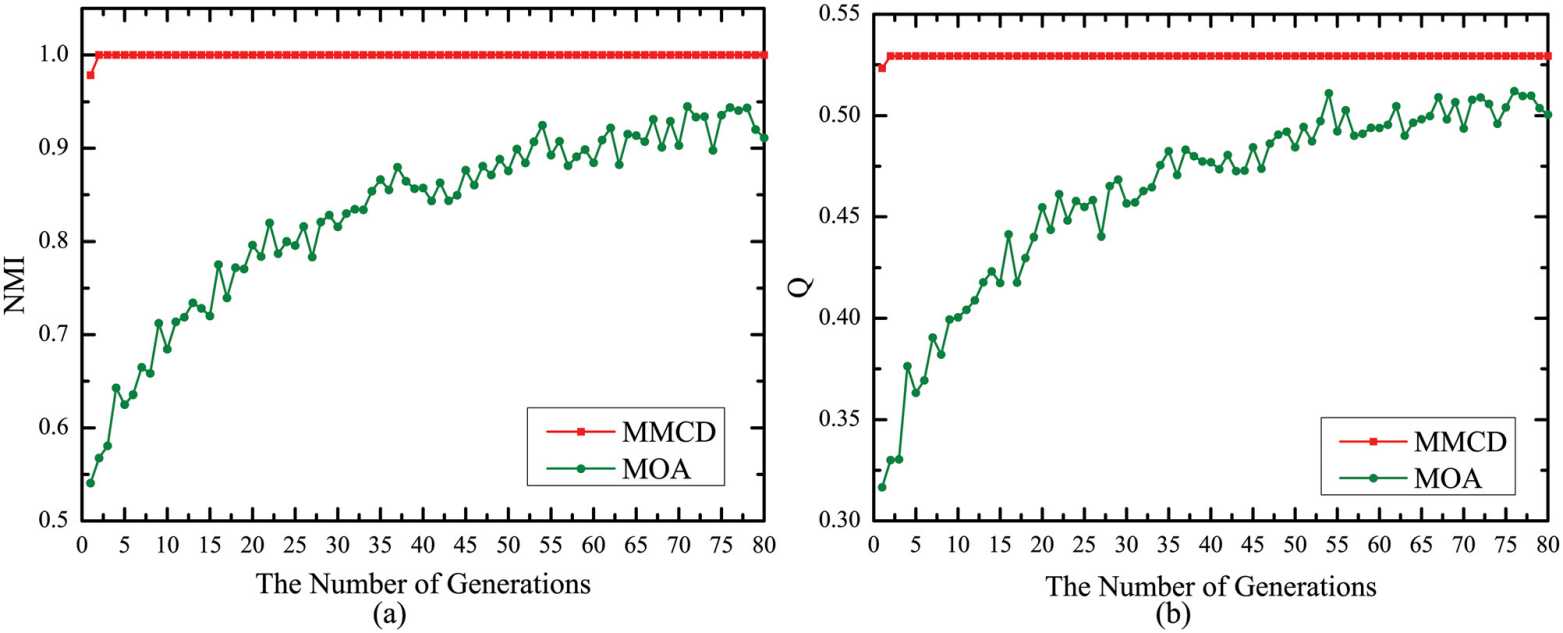
Best *NMI* and *Q* values averaged over 10 runs for MMCD and MOA. (a) Best *NMI* values with different generation numbers; (b) Best *Q* values with different generation numbers.

The maximum iteration number of local search procedure has been proved to have little influence on performance of MMCD. It is natural to come up with the doubt whether the local search procedure itself has influence on results of MMCD. In order to validate the effect of local search procedure, we develop a multi-objective optimization version of MMCD by removing the local search procedure and name it as MOA. MMCD and MOA with different generation numbers ranging from 1 to 80 with interval 1 are carried out on GN benchmark at *μ* = 0.2 ten times. The average best results of *NMI* and *Q* are displayed in Fig 6(a) and 6(b), respectively. It is obviously shown that MMCD can find the true partition and obtain the largest value of *NMI* and *Q* in just two generations. On the other hand, the increases of *NMI* and *Q* of MOA are much slower and it can’t find the true partition even after 80 generations. What’s more, the obvious fluctuation of the results of MOA indicates it is less stable than MMCD. In a word, the convergence results, the convergence speed and the stability of MMCD are all superior to MOA which demonstrates the positive effect of the local search procedure in algorithm.

### Comparison with Other Methods

In this section, performance of MMCD on finding single community structure is illustrated by comparing with other related methods. Three single objective optimization community detection methods named as CNM (A1), Louvain (A2), Infomap (A3), a single objective genetic algorithm GA-Net (A4), a single objective memetic algorithm Meme-Net (A5), two multi-objective genetic algorithms, MOGA-Net (A6) and MOCD (A7), and two simplified versions of MMCD named as MOA (A8) and LSA (A9) will be compared with MMCD (A10) on GN benchmark, LFR benchmark and a variety of real-world networks.

CNM [33] is a modularity optimization algorithm for community detection. Louvain [34] also optimize modularity but with a fast multistep greedy technique. Infomap [8] is based on the information theory to minimize a description length of information flow on network. All of three above methods are classic single objective optimization algorithms which are used to be compared with evolution based community detection algorithms.

GA-Net [21] is a representative single objective genetic algorithm which adopts a genetic algorithm to optimize a new objective function called community score. Meme-Net [14] is a single objective memetic algorithm which combines genetic algorithm with hill-climbing local search procedure to optimize the modularity. MOGA-Net [12] is a representative multi-objective evolutionary algorithm for community detection which optimizes two conflicting objectives, community score and community fitness. MOCD [13] is also a multi-objective evolutionary algorithm which optimizes two objectives similar to MMCD, i.e. two terms of modularity. All of four above methods are evolution based community detection algorithms from different perspectives.

As mentioned above, MOA is a simplified version of MMCD by removing the local search procedure from MMCD. It is used to validate the importance of local search procedure. On the other hand, LSA denotes a local search algorithm corresponding to local search part of MMCD. Since LSA can only optimize single objective function, we choose modularity as its objective function. In fact, LSA is the modularity-specialized LPA (LPAm) [24] which is used to test the importance of multi-objective scheme of MMCD.

**Fig 7:**
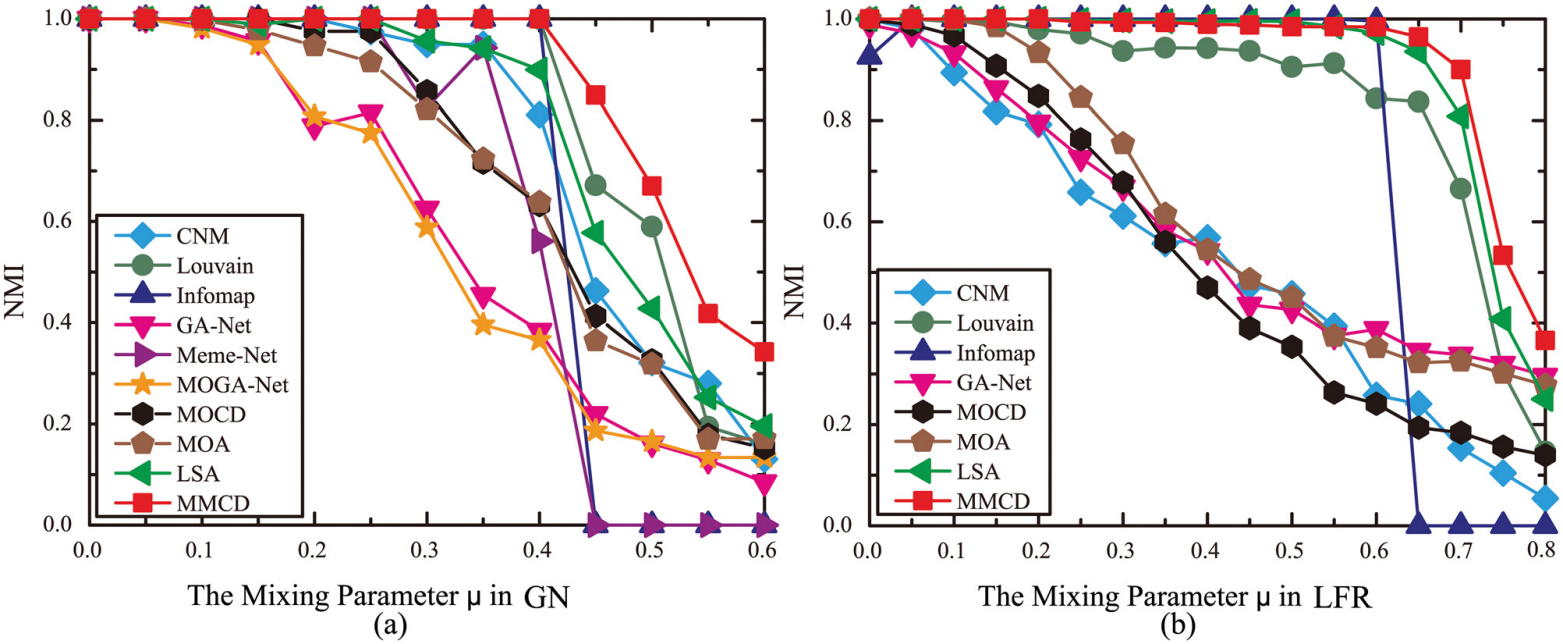
Best *NMI* values averaged over 10 runs for different algorithms on artificial datasets. (a) On GN benchmark dataset; (b) On LFR benchmark dataset. Meme-Net and MOGA-Net can’t give outputs within a given time (4 hours).

Firstly, all algorithms are compared on GN benchmark dataset. The experimental parameters of algorithms on GN benchmark dataset are listed in Table 5. Fig 7(a) summarizes the best *NMI* values averaged over 10 runs for different algorithms on GN benchmark dataset. When the mixing parameter *μ* is no bigger than 0.05, all algorithms can detect the true community structure. However, with the increase of *μ*, the performance of GA-Net, MOGA-Net, MOCD and MOA gradually decline. When the *μ* is no smaller than 0.25 and no bigger than 0.4, only Infomap, Louvain and MMCD can detect the true community structure. As the *μ* further increases, the network becomes vaguer and all algorithms can’t find the true partition. However, from the curves we can see that the MMCD still has the largest *NMI* values which indicates it is superior to the rest algorithms on GN benchmark datasets with *μ* bigger than 0.4. Note that the average NMI values of MOA, MOCD and MOGA-Net are much smaller than that of MMCD when *μ* is bigger than 0.25, though their maximum generation numbers are much larger than that of MMCD. This further demonstrates the positive importance of local search procedure in MMCD, as MOA, MOCD and MOGA-Net do not have local search procedure.

**Table 5:** Parameters of algorithms for GN and LFR benchmark datasets.

Algorithm	*Pop*		*Gmax*		*p*_*c*_	*p*_*m*_	*MI*	
	GN	LFR	GN	LFR			GN	LFR
GA-Net	100	200	100	200	0.8	0.2	—	—
Meme-Net	100	200	100	200	0.8	0.2	—	—
MOGA-Net	100	200	100	200	0.8	0.2	—	—
MOCD	100	200	100	200	0.8	0.2	—	—
MOA	100	200	100	200	—	0.01	—	—
LSA	—	—	—	—	—	—	10	20
MMCD	100	200	10	20	—	0.01	1	1

*Pop* represents the population size (it is maximum size of dominant population for MOA and MMCD), *Gmax* denotes the maximum generation number, *p*_*c*_ and *p*_*m*_ are the crossover and mutation probability, respectively. *MI* is the maximum iterations in local search procedure.—denotes that the value does not exist.

Since the GN benchmark cannot reflect some important features of real-world networks, another benchmark named as LFR [35] is also adopted to test different algorithms. The LFR benchmark is similar to real-world networks by introducing heterogeneity into degree and community size distributions of a network which are governed by power laws with exponents *τ*_1_ and *τ*_2_, respectively. Each node shares a fraction 1 − *μ* of edges with the nodes of its community and a fraction *μ* of edges with the rest of the network. *μ* is also called the mixing parameter. Network structure becomes fuzzier as *μ*’s value gets larger. In our experiments, 17 networks with the mixing parameter increasing from 0 to 0.8 with an interval of 0.05 are generated. Each network contains 1000 nodes and the community size ranges from 10 to 50. The averaged degree for each node is 20 and the max node degree is 50. *τ*_1_ = 2 and *τ*_2_ = 1.

The experimental parameters of algorithms for LFR benchmark dataset are listed in Table 5. Fig 7(b) summarizes the best *NMI* values averaged over 10 runs for different algorithms on LFR benchmark dataset. Infomap can’t detect the true community structure at *μ* = 0, but its *NMI* value achieves 1 after *μ* = 0 until *μ* = 0.55. MMCD and LSA detect the true community structure when the *μ* is no bigger than 0.2, and their *NMI* values are quite close to 1 until *μ* = 0.6. When *μ* surpasses 0.6, MMCD has the largest *NMI* value. However, two other multi-objective community detection methods, i.e. MOCD and MOA, have much poorer performance than MMCD when *μ* is bigger than 0.2. Since the main difference between them and MMCD is that they don’t have local search procedure, this illustrates the advantages of local search procedure in MMCD again.

Real-world networks may have different properties compared with artificial benchmark datasets, so all algorithms are further compared on a set of real-world networks. The description of each network is as follows.

Zachary’s karate club network (N1) [36] describes the friendships between 34 members of a karate club. The network was divided into two groups after a dispute between the administrator and the instructor.

Journal index network (N2) [37] consists of 40 journals as nodes from 4 different fields, i.e. physics, chemistry, biology and ecology. Edges exist between journals if at least one article from one journal cited an article in the other one during 2004.

Dolphin social network (N3) [38] consists of 62 bottlenose dolphins living in Doubtful Sound, Zealand. The ties between dolphin pairs were established by observation of statistically significant frequent association. The network naturally splits into two large groups.

Lesmis network (N4) [39] is a weighted network of coappearances of characters in Victor Hugo’s novel “Les Miserables”. The network is regarded as unweighted one by set the weight of all edges as 1 in this paper.

Polbooks network (N5) consists 105 nodes representing books about US politics sold by the online book seller Amazon.com. Edges represent frequent co-purchasing of books by the same buyers. Books were divided by Newman [40] into three groups according to their political alignments, i.e. liberal, neutral and conservative.

Word adjacency network (N6) [41] is the network of common words in the novel “David Copperfield” by Charles Dickens. Nodes represent the most commonly occurring adjectives and nouns. Edges connect any pair of words that occur in adjacent position in the text of the book.

American College Football network (N7) [1] represents American football games between Division IA colleges during regular season Fall 2000. Nodes represent teams and edges represent the regular games between two teams. The teams are grouped into 12 conferences.

SFI network (N8) [1] represents collaborations between scientists at the Santa Fe Institute during any part of calendar year 1999 or 2000. Edges connect any pair of scientists if they coauthored one or more articles during the same time period. The biggest component of the SFI graph with 118 nodes is used in the experiment.

Jazz musicians network (N9) [42] includes 198 bands that performed between 1912 and 1940. An edge between two bands is established if they have at least one musician in common. Neural network (N10) [43] represents the neural network of C. Elegans. Metabolic network (N11) [44, 45] represents metabolic system of C. Elegans. Email network (N12) [46] represents e-mail interchanges between members of the Univeristy Rovira i Virgili. Netscience network (N13) [41] records coauthorship of scientists working on network theory and experiments. Power network (N14) [43] represents the topology of the Western States Power Grid of the United States.

All the networks above are obtained from Internet [47–49]. The basic information of these real-world networks is shown in Table 6. All networks are divided into three groups according to their sizes, i.e., set of networks with nodes number smaller than 150 is group one (G1 = {N1, N2, N3, N4, N5, N6, N7, N8}), set of rest networks with nodes number smaller than 1000 is group two (G2 = {N9, N10, N11}), and the rest is group three (G3 = {N12, N13, N14}). The experimental parameters of algorithms for three network groups are listed in Table 7. Since MMCD converges faster, we adopt much smaller maximum generation number for it.

**Table 6:** The basic information of the real-world networks used in this paper.

Network	Nodes	Edges	⟨*k*⟩
N1	34	78	4.588
N2	40	189	9.450
N3	62	159	5.129
N4	77	254	6.597
N5	105	441	8.400
N6	112	425	7.589
N7	115	613	10.66
N8	118	200	3.390
N9	198	2742	27.70
N10	297	2345	15.79
N11	453	2025	8.940
N12	1133	5451	9.622
N13	1589	2742	3.451
N14	4941	6594	2.669

Nodes and Edges represent nodes’ number and edges’ number of network, respectively. ⟨*k*⟩ denotes the average degree of the network.

**Table 7:** Parameters of algorithms for real-world datasets.

Algorithm	*Pop*			*Gmax*			*p*_*c*_			*p*_*m*_			*MI*		
	G1	G2	G3	G1	G2	G3	G1	G2	G3	G1	G2	G3	G1	G2	G3
GA-Net	100	200	300	100	100	200	0.8	0.8	0.8	0.2	0.2	0.2	—	—	—
Meme-Net	100	200	300	100	100	200	0.8	0.8	0.8	0.2	0.2	0.2	—	—	—
MOGA-Net	100	200	300	100	100	200	0.8	0.8	0.8	0.2	0.2	0.2	—	—	—
MOCD	100	200	300	100	100	200	0.8	0.8	0.8	0.2	0.2	0.2	—	—	—
MOA	100	200	300	100	100	200	—	—	—	0.01	0.01	0.01	—	—	—
LSA	—	—	—	—	—	—	—	—	—	—	—	—	10	10	20
MMCD	100	200	300	10	20	40	—	—	—	0.01	0.01	0.01	1	1	1

*Pop* represents the population size (it is maximum size of dominant population for MOA and MMCD), *Gmax* denotes the maximum generation number, *p*_*c*_ and *p*_*m*_ are the crossover and mutation probability, respectively. *MI* is the maximum iterations in local search procedure. — denotes that the value does not exist.

For each network, we run all algorithms 10 times and record the maximum and average results and standard deviations. The comparison results of modularity are shown in Table 8. As CNM, Louvain and Infomap are deterministic algorithms, their standard deviations are 0 all the time which means they don’t have stability problem. Standard deviations of these three algorithms are removed since they don’t need to test stability performance. Bold number in each row denotes the best value in corresponding item. It can be seen from the Table 8 that there are 42 items in total for all networks. MMCD acquires the best values in 29 items. Specifically, MMCD has better or equal values in all criteria compared to GA-Net and Meme-Net which are two single objective evolutionary algorithms. Therein, Meme-Net is a single objective memetic algorithm which includes a local search procedure. This illustrates the superiority of multi-objective optimization strategy of MMCD. MMCD also has better or equal values in almost all criteria compared to MOGA-Net except in *Q*_*std*_ of N1, and it has better or equal performance on almost all networks compared to MOCD except on N1 and N8. What’s more, almost 90 percent of items of MOA are worse than MMCD and no item of MOA is better than MMCD. Since above three multi-objective optimization methods don’t include local search procedure, it demonstrates the advantages of the integrated local search procedure in MMCD. Specifically, larger *Q*_*max*_ and *Q*_*avg*_ values mean MMCD has better convergence ability and the smaller *Q*_*std*_ values show MMCD is more stable. As for rest four single objective heuristic methods, only Louvain is competitive with MMCD. The *Q*_*max*_ and *Q*_*avg*_ values of CNM on most networks are smaller than MMCD except on Netscience network and Power network. Similarly, the *Q*_*max*_ and *Q*_*avg*_ values on nearly all networks of Infomap are smaller than MMCD except on Netscience network. Finally, that the *Q*_*max*_ and *Q*_*avg*_ values of MMCD are larger than or equal to those of LSA on all networks and the *Q*_*std*_ values of MMCD are smaller than those of LSA except on Power network indicates that the evolutionary algorithm can help local search procedure escape from local optima and get better global solutions.

**Table 8:** The maximum, average and standard deviation of best modularity values (*Q*_*max*_, *Q*_*avg*_, *Q*_*std*_) obtained over 10 runs on fourteen real-word networks.

Network	Criteria	A1	A2	A3	A4	A5	A6	A7	A8	A9	A10
N1	*Q*_*max*_	0.3807	0.4188	0.4020	**0.4198**	0.4020	0.4156	**0.4198**	**0.4198**	**0.4198**	**0.4198**
	*Q*_*avg*_	0.3807	0.4188	0.4020	0.4084	0.3990	0.4156	**0.4198**	0.4188	0.3850	0.4191
	*Q*_*std*_	—	—	—	0.0087	0.0097	**0.0000**	**0.0000**	0.0024	0.0226	0.0015
N2	*Q*_*max*_	0.4596	**0.4783**	0.4567	**0.4783**	**0.4783**	**0.4783**	**0.4783**	**0.4783**	**0.4783**	**0.4783**
	*Q*_*avg*_	0.4596	**0.4783**	0.4567	0.4664	0.4675	**0.4783**	**0.4783**	0.4768	**0.4783**	**0.4783**
	*Q*_*std*_	—	—	—	0.0132	0.0114	**0.0000**	**0.0000**	0.0031	**0.0000**	**0.0000**
N3	*Q*_*max*_	0.4955	0.5188	0.5230	0.4950	0.5164	0.5253	**0.5285**	**0.5285**	0.5107	**0.5285**
	*Q*_*avg*_	0.4955	0.5188	0.5230	0.4719	0.4989	0.5183	0.5265	0.5244	0.5001	**0.5269**
	*Q*_*std*_	—	—	—	0.0137	0.0210	0.0070	**0.0014**	0.0032	0.0099	**0.0014**
N4	*Q*_*max*_	0.5006	0.5583	0.5513	0.5402	0.5397	0.5476	**0.5600**	0.5537	0.5357	**0.5600**
	*Q*_*avg*_	0.5006	**0.5583**	0.5513	0.5138	0.5247	0.5426	0.5570	0.5482	0.5318	0.5578
	*Q*_*std*_	—	—	—	0.0250	0.0171	0.0060	0.0024	0.0029	0.0042	**0.0023**
N5	*Q*_*max*_	0.5020	0.5268	0.5268	0.5076	0.5248	0.5267	**0.5272**	0.5271	0.5185	**0.5272**
	*Q*_*avg*_	0.5020	0.5268	0.5268	0.4910	0.5151	0.5222	0.5265	0.5160	0.5014	**0.5271**
	*Q*_*std*_	—	—	—	0.0152	0.0108	0.0058	0.0013	0.0017	0.0138	**0.0001**
N6	*Q*_*max*_	0.2953	0.2886	0.0333	0.2199	0.1198	0.1746	0.2810	0.2526	0.2782	**0.3082**
	*Q*_*avg*_	0.2953	0.2886	0.0333	0.1507	0.0166	0.1280	0.2634	0.2380	0.2679	**0.3002**
	*Q*_*std*_	—	—	—	0.0315	0.0366	0.0229	0.0110	0.0094	0.0109	**0.0036**
N7	*Q*_*max*_	0.5772	0.6043	0.6005	0.5798	0.6031	0.5960	0.6021	0.6044	0.6031	**0.6046**
	*Q*_*avg*_	0.5772	**0.6043**	0.6005	0.5602	0.5895	0.5609	0.5874	0.5978	0.5863	0.6040
	*Q*_*std*_	—	—	—	0.0171	0.0172	0.0184	0.0080	0.0067	0.0158	**0.0006**
N8	*Q*_*max*_	0.7335	**0.7506**	0.6509	0.6060	0.7212	0.7484	**0.7506**	0.7466	0.6101	0.7487
	*Q*_*avg*_	0.7335	**0.7506**	0.6509	0.5779	0.7076	0.7424	0.7500	0.7382	0.5671	0.7435
	*Q*_*std*_	—	—	—	0.0200	0.0137	0.0048	**0.0005**	0.0077	0.0215	0.0031
N9	*Q*_*max*_	0.4389	**0.4451**	0.4423	0.4049	0.4386	0.2952	0.4374	0.4430	0.4448	**0.4451**
	*Q*_*avg*_	0.4389	**0.4451**	0.4423	0.2936	0.3717	0.2929	0.4279	0.4404	0.4375	0.4449
	*Q*_*std*_	—	—	—	0.0412	0.0779	0.0084	0.0060	0.0023	0.0068	**0.0002**
N10	*Q*_*max*_	0.3692	0.3835	0.3917	0.2808	0.3786	0.2433	0.3737	0.3710	0.3913	**0.4061**
	*Q*_*avg*_	0.3692	0.3835	0.3917	0.2070	0.3096	0.1370	0.3440	0.3622	0.3647	**0.4046**
	*Q*_*std*_	—	—	—	0.0627	0.0567	0.0691	0.0148	0.0075	0.0165	**0.0017**
N11	*Q*_*max*_	0.4061	0.4320	0.4194	0.3082	\	0.2728	0.3931	0.3825	0.3993	**0.4415**
	*Q*_*avg*_	0.4061	0.4320	0.4194	0.2963	\	0.2217	0.3845	0.3715	0.3905	**0.4362**
	*Q*_*std*_	—	—	—	0.0075	\	0.0272	0.0062	0.0099	0.0059	**0.0037**
N12	*Q*_*max*_	0.5130	0.5720	0.5340	0.3122	\	0.3475	0.4771	0.5219	0.5170	**0.5749**
	*Q*_*avg*_	0.5130	0.5720	0.5340	0.2920	\	0.3076	0.4433	0.4921	0.4930	**0.5725**
	*Q*_*std*_	—	—	—	0.0116	\	0.0215	0.0218	0.0223	0.0156	**0.0018**
N13	*Q*_*max*_	0.9555	**0.9592**	0.9581	\	\	\	0.9410	0.9394	0.8691	0.9394
	*Q*_*avg*_	0.9555	**0.9592**	0.9581	\	\	\	0.9269	0.9347	0.8629	0.9347
	*Q*_*std*_	—	—	—	\	\	\	0.0071	**0.0032**	0.0039	**0.0032**
N14	*Q*_*max*_	0.9341	**0.9348**	0.8175	\	\	\	0.7372	0.6799	0.6269	0.8270
	*Q*_*avg*_	0.9341	**0.9348**	0.8175	\	\	\	0.7308	0.6721	0.6230	0.8224
	*Q*_*std*_	—	—	—	\	\	\	0.0039	0.0043	**0.0021**	0.0030

— denotes the value is removed. \ denotes the corresponding algorithms can’t give outputs within a given time (3 hours). Bold number in each row denotes the best value in corresponding item.

Among real-world networks, Karate, Journal, Dolphins, Polbooks and Football networks have ground-truth community structures. The comparison results of *NMI* on these five networks are shown in Table 9. Standard deviations of three deterministic algorithms are removed as well. Bold number in each row denotes the best value in corresponding item. From the Table 9 it is obviously shown that the *NMI*_*max*_ and *NMI*_*avg*_ values of single objective optimization algorithms (i.e. CNM, Louvain, Infomap, GA-Net, Meme-Net and LSA) on most real-world networks are much lower than those of multi-objective optimization methods with only a few exceptions. The reason is that they try to optimize only one objective function (modularity, map equation, modularity density, etc.) and get only single solution. If the objective function can’t exactly describe the network properties, the sole solution will not match the true partition. This observation demonstrates the drawbacks of single objective optimization methods mentioned in the previous section. The advantage of local search procedure is further proven by comparing MMCD with MOA. All *NMI*_*avg*_ values of MOA except that on Karate network are smaller than those of MMCD and all *NMI*_*std*_ values of MOA are larger than those of MMCD which illustrate the wonderful effects of local search procedure on convergence ability and stability of MMCD. Besides, MMCD is comparable with MOGA-Net and MOCD on *NMI* values.

**Table 9:** The maximum, average and standard deviation of best *NMI* values (*NMI*_*max*_, *NMI*_*avg*_, *NMI*_*std*_) obtained over 10 runs on five real-word networks with known true partition.

Network	Criteria	A1	A2	A3	A4	A5	A6	A7	A8	A9	A10
N1	*NMI*_*max*_	0.5767	0.4469	0.5925	0.5657	**1.0000**	**1.0000**	0.8361	**1.0000**	0.5235	**1.0000**
	*NMI*_*avg*_	0.5767	0.4469	0.5925	0.5272	0.6333	**1.0000**	0.8361	0.9039	0.4781	0.8689
	*NMI*_*std*_	—	—	—	0.0339	0.1289	**0.0000**	**0.0000**	0.1221	0.0318	0.0691
N2	*NMI*_*max*_	0.8791	**1.0000**	0.7500	**1.0000**	**1.0000**	**1.0000**	**1.0000**	**1.0000**	**1.0000**	**1.0000**
	*NMI*_*avg*_	0.8791	**1.0000**	0.7500	0.9691	0.9250	**1.0000**	**1.0000**	0.9354	**1.0000**	**1.0000**
	*NMI*_*std*_	—	—	—	0.0542	0.1208	**0.0000**	**0.0000**	0.0401	**0.0000**	**0.0000**
N3	*NMI*_*max*_	0.4378	0.3645	0.3655	0.3440	0.4556	**1.0000**	0.8809	0.8809	0.3333	**1.0000**
	*NMI*_*avg*_	0.4378	0.3645	0.3655	0.2853	0.3857	**0.9808**	0.8809	0.6358	0.2960	0.9167
	*NMI*_*std*_	—	—	—	0.0270	0.0469	0.0606	**0.0000**	0.1413	0.0214	0.0575
N5	*NMI*_*max*_	0.5079	0.4759	0.4759	0.3595	0.4363	0.5979	**0.6065**	0.6007	0.3720	0.5765
	*NMI*_*avg*_	0.5079	0.4759	0.4759	0.3330	0.3858	**0.5885**	0.5763	0.5060	0.3273	0.5716
	*NMI*_*std*_	—	—	—	0.0184	0.0367	0.0104	0.0132	0.0510	0.0197	**0.0056**
N7	*NMI*_*max*_	0.6698	0.8529	0.9232	0.9268	0.8955	0.9065	0.9158	0.8847	0.9239	**0.9397**
	*NMI*_*avg*_	0.6698	0.8529	0.9232	0.8630	0.8455	0.8358	0.8812	0.8154	0.8858	**0.9261**
	*NMI*_*std*_	—	—	—	0.0418	0.0491	0.0362	0.0221	0.0504	0.0410	**0.0053**

— denotes the value is removed. Bold number in each row denotes the best value in corresponding item.

### Hierarchical Community Structures of Network

Not only MMCD can figure out high quality community structures, but also the population nature of its solutions can help find multi-resolution community structures in one run. Firstly, a simple hierarchical extension of the GN benchmark is adopted as hierarchical artificial network with built-in hierarchical community structures [50]. The network consists of 256 nodes which are arranged in 16 communities with 16 nodes each. The 16 communities are further grouped into four supercommunities, each of which has four smaller communities. The structure with 16 communities is regarded as level 1 community structure and the one with four supercommunities is regarded as level 2 community structure. Communities in level 1 and level 2 structures are called level 1 and level 2 communities, respectively. Each node shares average of 10 edges with the nodes in its level 1 community and 5 edges with nodes in three other level 1 communities in its level 2 community. In addition, each node has average of 2 edges with the rest of the network.

**Fig 8:**
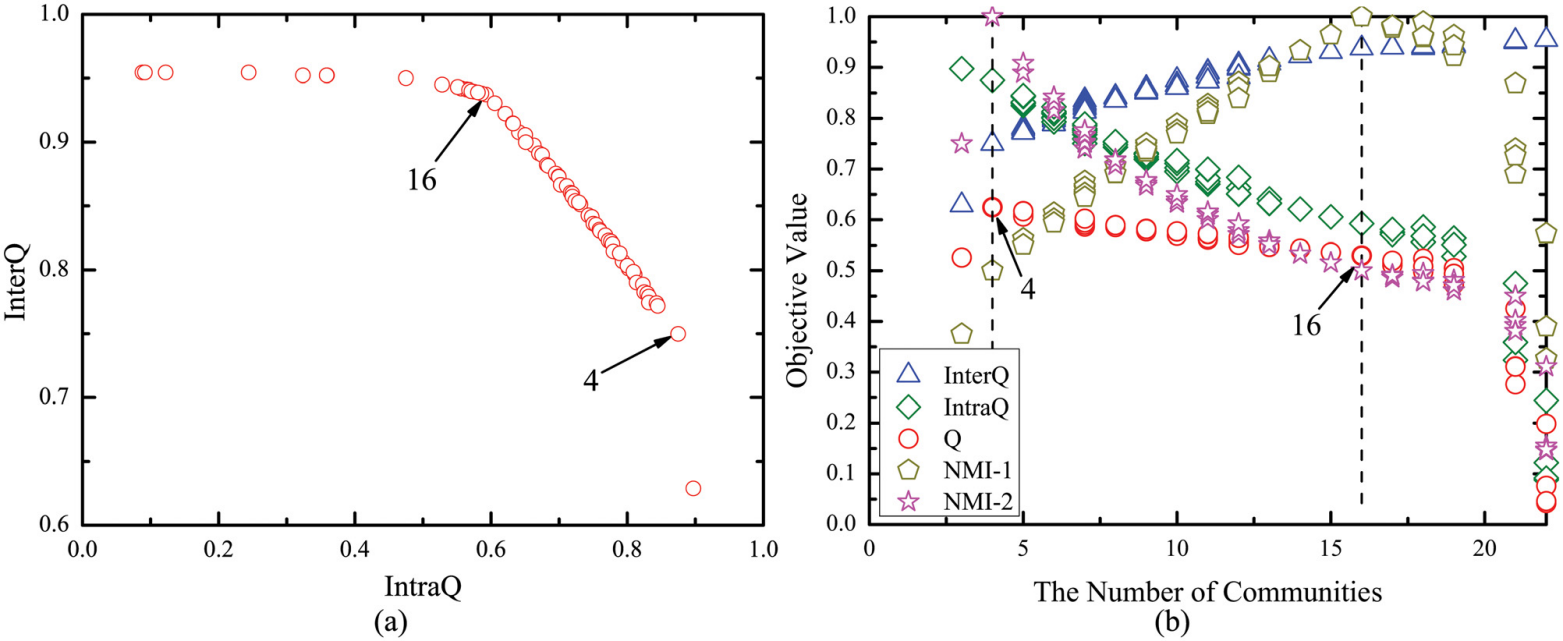
Results of MMCD on hierarchical GN benchmark. (a) Nondominated front of final solution population; (b) Relationships between some objective values and the number of communities.

**Fig 9:**
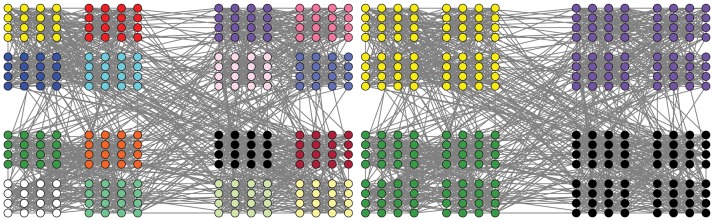
Two representative community structures obtained by MMCD on hierarchical GN benchmark.

The MMCD is carried out on hierarchical GN benchmark once. The nondominated front of final solution population is shown in Fig 8(a). By analyzing the solution population, two community structure levels are both exactly found out. The arrow symbols with numbers of community in Fig 8(a) and 8(b) indicate the solutions corresponding to two community partition levels. Two representative community structures with 16 smaller communities and 4 larger communities found by MMCD are plot in Fig 9. In addition to two representative community structure levels, we further analyze the other solutions obtained by MMCD. The relationships between some objective values and the number of communities are shown in Fig 8(b). *NMI* − 1 and *NMI* − 2 denotes *NMI* values between the partitions found by MMCD and the true level 1 and level 2 community structures, respectively. It is shown that level 2 community structure has the largest modularity (*Q*) value which indicates that single modularity optimization algorithms may fail to figure out the level 1 community structure. In fact, the modularity of level 1 community structure even is not the second largest one. The modularity values of all partitions with community numbers between 4 and 16 are close to 0.6. These partitions are formed by merging several smaller level 1 communities together, so they are all reasonable partitions besides the level 1 and level 2 community structures. However, for those partitions with low modularity values, some tightly connected level 1 communities are split. They are unreasonable community structures. So modularity can be used to select reasonable partitions from the solution population obtained by MMCD. *IntraQ* and *InterQ* are two objective functions of MMCD. From the diagram, we can see that *IntraQ* decreases monotonically with the number of communities while *InterQ* increases monotonically. This observation agrees with the property mentioned in the previous section that the maximization of *IntraQ* and *InterQ* tend to find communities of opposite sizes. The *NMI* − 1 value and *NIM* − 2 value also have an opposite change trends along community numbers within range from 4 to 16 which means higher similarity with level 1 community structure will inevitably leads to lower similarity with level 2 community structure at the range [4, 16].

**Fig 10:**
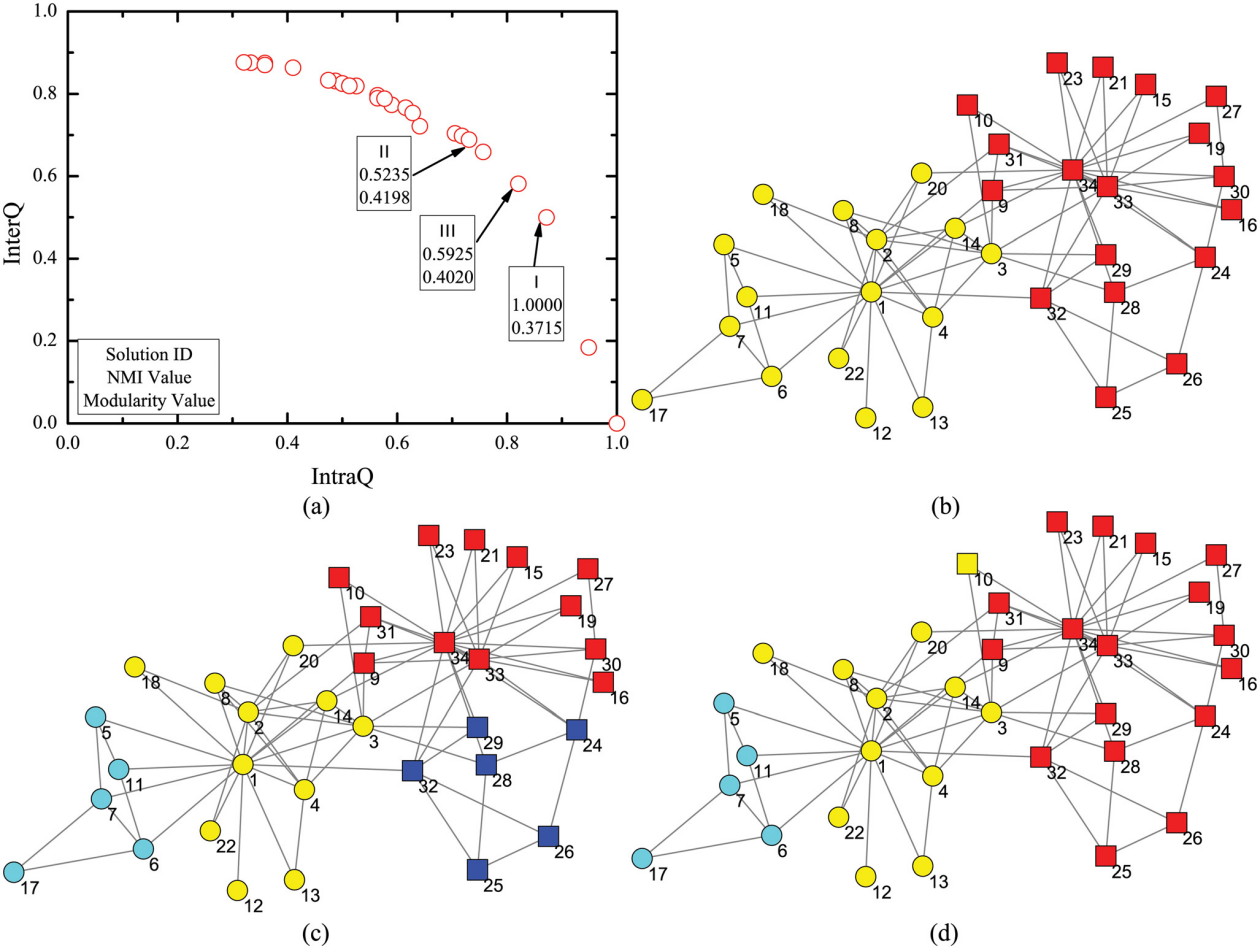
Results of MMCD on Karate network. (a) Nondominated front; (b)-(d) correspond to three solutions labeled as I-III in nondominated front, respectively. Squares and circles represent true communities. Different colors denote communities obtained by MMCD.

We further illustrate the advantages of MMCD for identifying multi-resolution structures on real-world networks. The MMCD is carried out on each real-world network once. Fig 10(a) shows the nondominated front of MMCD on Karate network. Fig 10(b)–10(d) correspond to three solutions labeled as I-III in nondominated front, respectively. Fig 10(b) divides network into two communities exactly as true partition and its modularity value is 0.3715. The maximum modularity value found by MMCD is 0.4918 whose corresponding community structure is in Fig 10(c). In fact, Fig 10(c) further divides each community in Fig 10(b) into two smaller ones. The partition with the maximum modularity may not correspond to true partition in reality, so algorithms only optimizing modularity may fail to find out the true partition of a network. Fig 10(d) is another structure obtained with three communities whose modularity is 0.4020.

**Fig 11:**
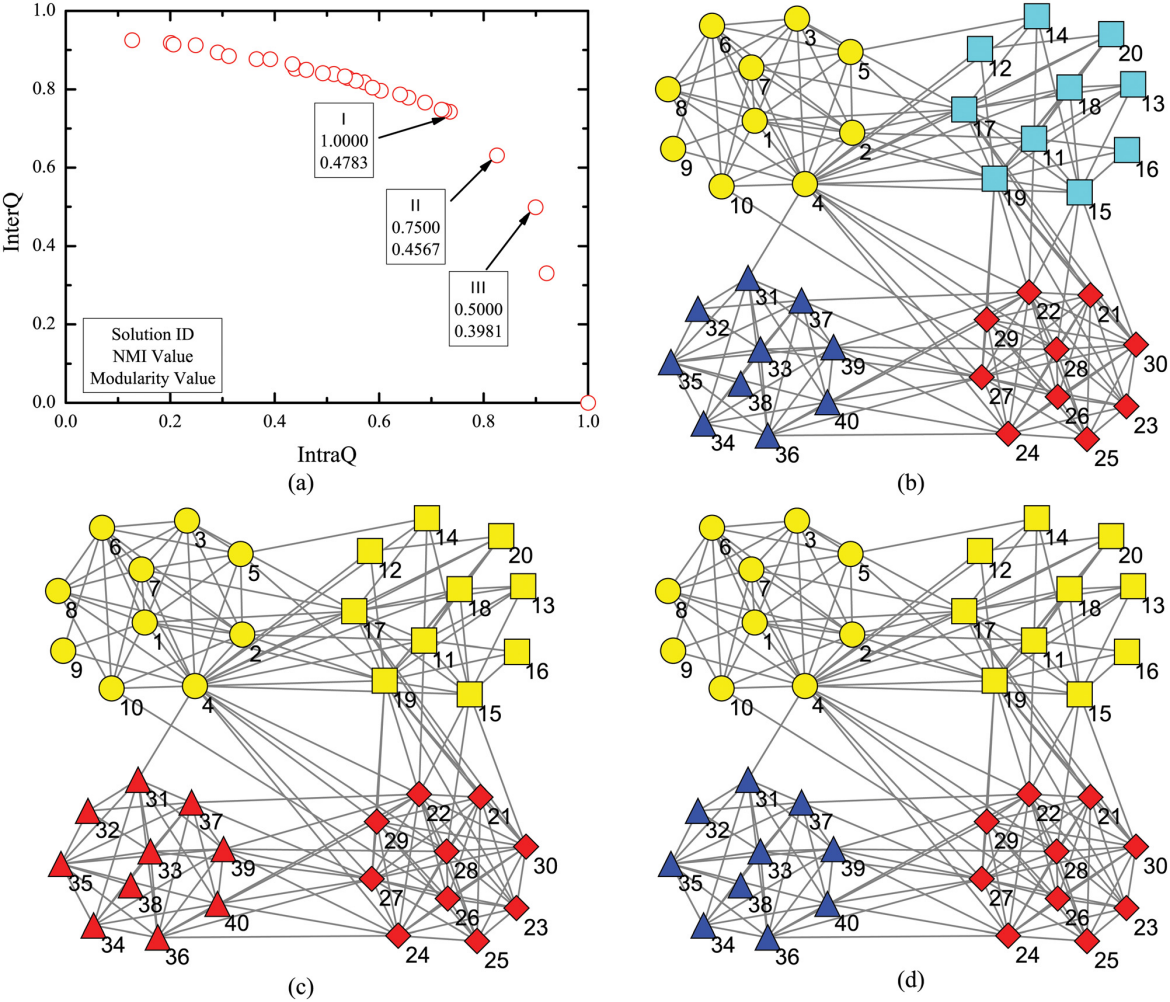
Results of MMCD on Journal network. (a) Nondominated front; (b)-(d) correspond to three solutions labeled as I-III in nondominated front, respectively. Circles, Squares, diamonds and triangles represent physics, chemistry, biology and ecology journals in true partition, respectively. Different colors denote communities obtained by MMCD.

Fig 11(a) displays the nondominated front of Journal network. Community structure in Fig 11(b) corresponds to solution I in Fig 11(a) which has the maximal *NMI* value and maximal modularity value at the same time. Fig 11(c) and 11(d) are two other partitions corresponding to solution II and III, respectively. The upper two communities in Fig 11(b) are mainly about physics and chemistry respectively, and the lower two communities are mainly about biology and ecology respectively. From the perspective of relations between disciplines, the upper two communities have stronger connections and can be merged into a larger community, and it is the same to the lower two communities.

**Fig 12:**
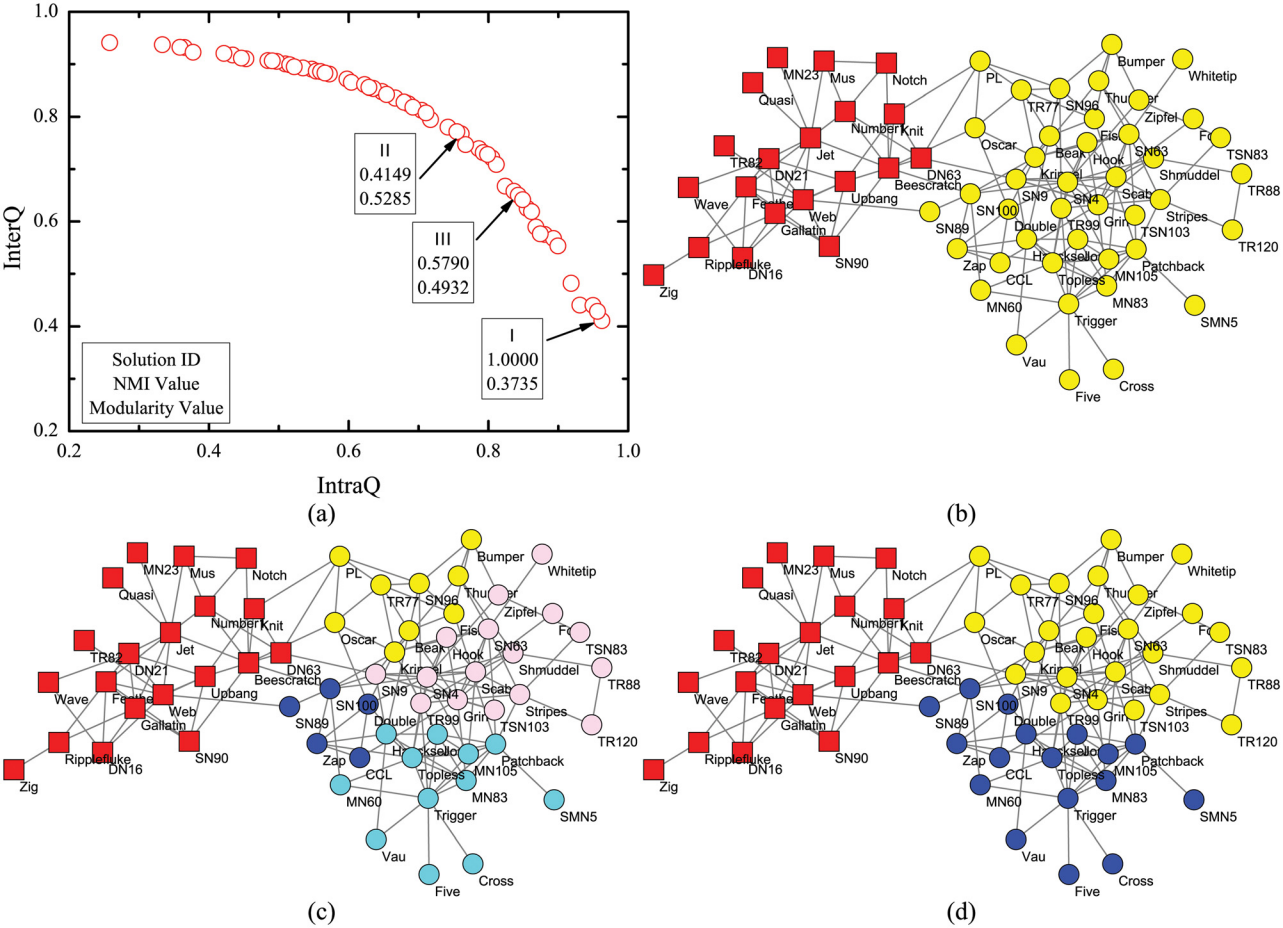
Results of MMCD on Dolphins network. (a) Nondominated front; (b)-(d) correspond to three solutions labeled as I-III in nondominated front, respectively. Squares and circles represent true communities. Different colors denote communities obtained by MMCD.

Fig 12(a) shows the nondominated front obtained on Dolphins network. Partitions corresponding to maximal *NMI* value (solution I) and maximal modularity value (solution II) are illustrated in Fig 12(b) and 12(c). Fig 12(c) divides the larger community in Fig 12(b) into four smaller ones. Fig 12(d) is another obtained solution with three communities corresponding to solution III in Fig 12(a) whose modularity value is 0.4932. The structure of Fig 12(d) is just between the structures of Fig 12(b) and Fig 12(c). It divides the larger community in Fig 12(b) into two smaller ones and merges four smaller communities in Fig 12(c) into two larger ones.

**Fig 13:**
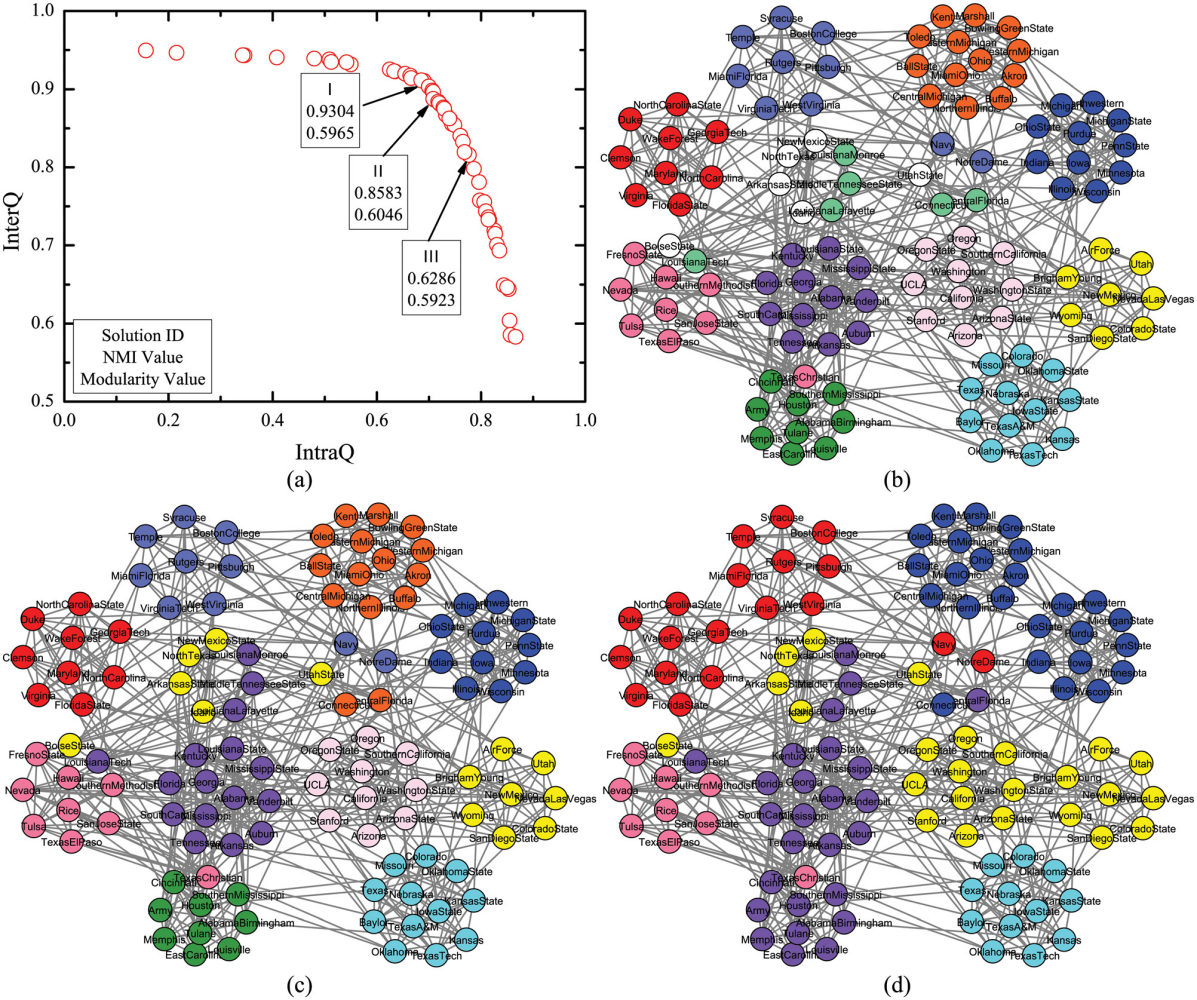
Results of MMCD on Football network. (a) Nondominated front; (b)-(d) correspond to three solutions labeled as I-III in nondominated front, respectively. Groups of nodes gathered together represent true communities. Different colors denote communities obtained by MMCD.

The nondominated front obtained on Football network is shown in Fig 13(a). Groups of nodes gathered together represent true communities of Football network in Fig 13(b)–13(d). MMCD do not obtain the exactly true partition. The maximal *NMI* value obtained in one run is 0.9304 of solution I and the corresponding partition is shown in Fig 13(b). The number of communities in Fig 13(b) is 12 which is the same as true partition, however it misclassifies several nodes in two small communities in the middle of the network because such two communities are loosely connected inside themselves. Fig 13(c) shows the partition corresponding to solution II with the maximal modularity. Two small communities in the middle of the network disappear and their nodes are included into other communities. Solution III also has relative large modularity value and its community structure is illustrated in Fig 13(d). It has six communities. The main difference between solution III and two previous partitions is that it merges eight communities into four larger ones, each of which roughly consists of two smaller communities. By examining the names of football teams in the network carefully, some interesting phenomena are discovered. It is reasonable to assume that the names of teams reflect the location of them, e.g., Utah in yellow community in Fig 13(c) is in Utah of America. Based on this assumption, it can be observed that teams in each community detected by MMCD are mainly from the states near each other with only a few exceptions. For example, teams named Utah, NevadaLasVegas, NewMexico, ColoradoState, Wyoming, SanDiegoState, NewMexicoState, UtahState and so on in yellow community in Fig 13(c) are mainly from the southwest region of USA according to their names. Teams named WashingtonState, Washington, Oregon, OregonState, UCLA, California, ArizonaState and so on in pink community in Fig 13(c) are mainly from the pacific region of USA. In fact, the pacific region is adjacent to southwest region in America, so it is reasonable that the yellow community and pink community in Fig 13(c) are merged together in Fig 13(d). Other communities have the similar phenomena. This observation agrees with the common schedule arrangements of sports such as Divisions in National Basketball Association (NBA).

## Conclusions

In this paper, we formulate community detection as a multi-objective optimization problem which regards the two contradictory parts of modularity function as two objective functions. In order to optimize such two objective functions effectively, a multi-objective memetic algorithm named as MMCD is proposed. MMCD combines multi-objective evolutionary mechanism with a local search procedure which helps algorithm to locate better solutions more effectively. An effective local search procedure is designed by addressing three issues, i.e., determining initial individuals, defining appropriate fitness function and designing effective local search strategy. The extensive experiments on both artificial and real-world networks demonstrate the advantages of MMCD from three aspects. The integrated local search procedure is proven to speed up the convergence to optimal solutions and make the algorithm more stable. What’s more, experiments show that MMCD not only can find good community structures effectively, but also can figure out hierarchical structures which are useful to analyze networks in multi-resolution levels. Some meaningful extensions can be made to MMCD in the future. There are only two objective functions considered in MMCD. When more objective functions are incorporated, the nondominated front will be much more complicated. How to calculate the normal line direction of nondominated front effectively is still an open question.
